# Probiotic *Bifidobacterium breve* Induces IL-10-Producing Tr1 Cells in the Colon

**DOI:** 10.1371/journal.ppat.1002714

**Published:** 2012-05-31

**Authors:** Seong Gyu Jeon, Hisako Kayama, Yoshiyasu Ueda, Takuya Takahashi, Takashi Asahara, Hirokazu Tsuji, Noriko M. Tsuji, Hiroshi Kiyono, Ji Su Ma, Takashi Kusu, Ryu Okumura, Hiromitsu Hara, Hiroki Yoshida, Masahiro Yamamoto, Koji Nomoto, Kiyoshi Takeda

**Affiliations:** 1 Laboratory of Immune Regulation, Department of Microbiology and Immunology, Graduate School of Medicine, Osaka University, Suita, Osaka, Japan; 2 WPI Immunology Frontier Research Center, Osaka University, Suita, Osaka, Japan; 3 Core Research for Evolutional Science and Technology, Japan Science and Technology Agency, Saitama, Japan; 4 Yakult Central Institute for Microbiological Research, Kunitachi, Tokyo, Japan; 5 Age Dimension Research Center, National Institute of Advanced Industrial Science and Technology (AIST), Tsukuba, Ibaraki, Japan; 6 Division of Mucosal Immunology, Department of Microbiology and Immunology, Institute of Medical Science, University of Tokyo, Tokyo, Japan; 7 Division of Molecular and Cellular Immunoscience, Department of Biomolecular Sciences, Faculty of Medicine, Saga University, Nabeshima, Saga, Japan; Yale University, United States of America

## Abstract

Specific intestinal microbiota has been shown to induce Foxp3^+^ regulatory T cell development. However, it remains unclear how development of another regulatory T cell subset, Tr1 cells, is regulated in the intestine. Here, we analyzed the role of two probiotic strains of intestinal bacteria, *Lactobacillus casei* and *Bifidobacterium breve* in T cell development in the intestine. *B. breve*, but not *L. casei*, induced development of IL-10-producing Tr1 cells that express cMaf, IL-21, and Ahr in the large intestine. Intestinal CD103^+^ dendritic cells (DCs) mediated *B. breve*-induced development of IL-10-producing T cells. CD103^+^ DCs from *Il10* ^−/−^, *Tlr2* ^−/−^, and *Myd88* ^−/−^ mice showed defective *B. breve*-induced Tr1 cell development. *B. breve*-treated CD103^+^ DCs failed to induce IL-10 production from co-cultured *Il27ra* ^−/−^ T cells. *B. breve* treatment of *Tlr2* ^−/−^ mice did not increase IL-10-producing T cells in the colonic lamina propria. Thus, *B. breve* activates intestinal CD103^+^ DCs to produce IL-10 and IL-27 via the TLR2/MyD88 pathway thereby inducing IL-10-producing Tr1 cells in the large intestine. Oral *B. breve* administration ameliorated colitis in immunocompromised mice given naïve CD4^+^ T cells from wild-type mice, but not *Il10* ^−/−^ mice. These findings demonstrate that *B. breve* prevents intestinal inflammation through the induction of intestinal IL-10-producing Tr1 cells.

## Author Summary

Unlike induction of Foxp3^+^ regulatory T cell development, it remains unclear how intestinal environmental factors regulate development of another regulatory T cell subset, Tr1 cells that produce IL-10. In this study, we reveal that a probiotic strain, *Bifidobacterium breve* induces IL-10-producing Tr1 cells that express c-Maf, IL-21, and Ahr via activation of intestinal CD103^+^ DCs in the large intestine. Using several gene-targeted mice, we show that *B. breve*-induced development of IL-10-producing Tr1 cells is dependent on DC secretion of IL-10 and 27 via a TLR2/MyD88 pathway. We finally show that *B. breve* ameliorated T cell-dependent colitis in immunocompromised mice via T cell production of IL-10. These findings demonstrate that *B. breve* maintains intestinal homeostasis through the induction of intestinal IL-10-producing Tr1 cells.

## Introduction

Recent advances in metagenomic analysis of intestinal bacteria have revealed that inflammatory bowel diseases (IBD) is associated with dysbiosis in the intestinal microflora [1], [2], [3]. In support of these human studies, analysis of mice lacking NLRP6 has revealed that altered composition of intestinal symbiotic bacteria contributes to the pathogenesis of colitis [4]. Probiotics, live microorganisms which confer a health benefit on the host when administered in appropriate amounts, have been used for the treatment of IBD [5]–[8]. Probiotics have been shown to modulate the intestinal symbiotic bacteria leading to the maintenance of intestinal homeostasis [9], [10]. Modulation of microbiota by probiotics has been shown to be elicited by antagonizing pathogenic bacteria through the reduction of luminal pH, inhibition of bacterial adherence, or production of anti-microbial molecules [8]. Probiotics have also been shown to enhance barrier functions of intestinal epithelial cells [11]. Thus, several mechanisms for the cross-talk between probiotics and the host have been postulated.

Recent accumulating evidence has indicated that intestinal commensal microbiota has a great influence on the host intestinal immune system [12]–[14]. Commensal microbiota has been shown to induce IgA-mediated responses and development of Th1/Th17 effector T cells as well as regulatory T (Treg) cells [15]–[17]. More recently, a specific microbiota that induces development of Th17 cells or Treg cells has been demonstrated. Segmented filamentous bacteria (SFB), which have been previously shown to induce IgA-producing cells in the small intestine, were shown to induce Th17 cell development in the small intestine of mice [18], [19]. A human symbiotic bacterium, *Bacteroides fragilis* has been shown to mediate Toll-like receptor 2 (TLR2)-dependent development of Foxp3^+^ Treg cells in the large intestine [20]–[22]. *Clostridium* species mediate TLR-independent induction of Foxp3^+^ Treg cells in the large intestine [23]. Thus, several selective intestinal bacteria promote development of intestinal T cells via distinct mechanisms. Most recently, microbiota-dependent induction of Foxp3^+^ Treg cells has been shown to be required for the establishment of intestinal CD4^+^ T cell homeostasis [24]. Additionally, commensal microbiota has been shown to educate Foxp3^+^ Treg cells to acquire the antigen-specific repertoires of their T cell receptors [25]. Probiotics have also been shown to directly modulate the host immune system, especially the induction of Foxp3^+^ Treg or TGF-β-bearing Treg cell development [26]–[29]. Thus, several mechanisms for intestinal bacteria-dependent development of Foxp3^+^ Treg cells have been postulated.

Intestinal homeostasis is maintained by regulatory T cell populations consisting of two major CD4^+^ T cell subsets; Foxp3^+^ Treg cells and IL-10-producing type 1 regulatory T (Tr1) cells [30]. Tr1 cells modulate immune responses via mechanisms distinct from those used by Foxp3^+^ Treg cells [31]. Indeed, Tr1 cells do not express the master Treg transcription factor Foxp3, and are induced by distinct cytokines such as IL-10 and IL-27 [32], [33]. Tr1 cells are abundant in the intestinal lamina propria [34], yet it remains unclear how Tr1 cells develop in the intestine.

In this study, we analyzed the effect of two probiotic strains, *Bifidobacterium breve* and *Lactobacillus casei*, on intestinal T cells responses. Oral administration of *B. breve*, but not *L. casei*, resulted in increased IL-10 production from colonic CD4^+^ T cells, without enhancing Foxp3 expression. *B. breve*-induced IL-10-producing CD4^+^ T cells possessed properties of Tr1 cells, as evidenced by expression of *cMaf, Il21*, and *Ahr*. *B. breve*-dependent Tr1 cell induction was mediated by intestinal CD103^+^ dendritic cells via TLR2/MyD88-dependent production of IL-10 and IL-27. *B. breve* administration ameliorated intestinal inflammation in immunocompromised mice transferred with naïve CD4^+^ T cells in an IL-10-dependent manner. These findings establish the mechanisms for Tr1 cell induction by the probiotic *B. breve*, which modulates the host immune responses.

## Results

### *B. breve* induces IL-10-producing CD4^+^ T cell in the colon

**Figure 1:**
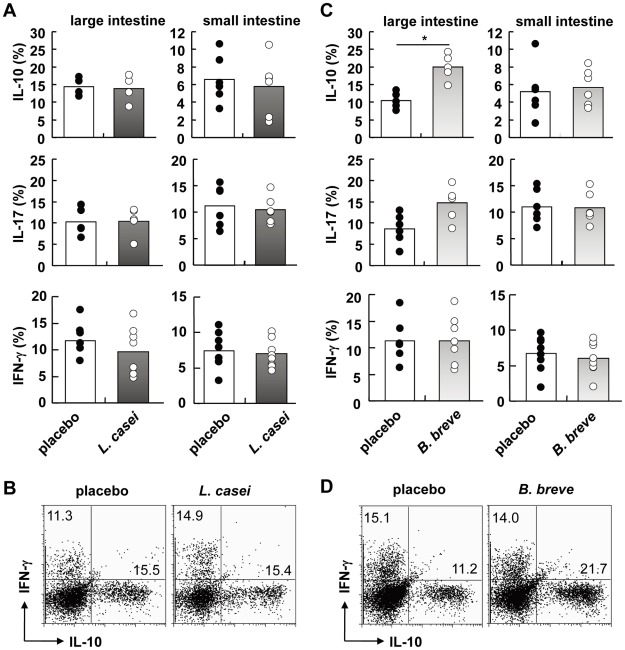
Induction of IL-10-producing CD4^+^ T cells by *B. breve* in the colonic lamina propria. 6-week-old C57BL/6 mice were fed *L. casei* or *B. breve* or placebo daily (each, 1×10^9^) by oral gavage for 3 months (n = 8). Intestinal lamina propria lymphocytes were analyzed for cytokine production by flow cytometry. Percentages of IL-10-, IL-17-, and IFN-γ-producing CD4^+^ T cells of mice administered with *L. casei* (**A**) or *B. breve* (**C**) were shown. *P<0.05. (**B**, **D**) Representative FACS dot plots showing production of IL-10 and IFN-γ gated on colonic CD4^+^ T cells in the indicated mice.

*Lactobacillus casei* strain Shirota and *Bifidobacterium breve* Yakult strain have been proven to be beneficial for the treatment of several diseases such as diabetes mellitus, arthritis and inflammatory bowel diseases [35]–[40]. In order to analyze the effect of these probiotic strains on the intestinal homeostasis, we orally treated C57BL/6 mice with *L. casei* and *B. breve* (10^9^ bacteria each) for 3 months. We first analyzed fecal microbiota using both quantitative PCR and reverse transcription-quantitative PCR methods targeting rDNA and rRNA, respectively [41]. Administration of *L. casei* and *B. breve* did not induce a significant change in the number and composition of microbiota (Text S1, Table S1). Because several microbiota have been shown to induce differentiation of intestinal CD4^+^ T cells [17], we analyzed production of IL-10, IL-17, and IFN-γfrom CD4^+^ T cells in the small intestine and large intestine of mice orally treated with *L. casei* and *B. breve*. The number of IL-10-, IL-17-, and IFN-γ-producing T cells in both the small intestine and the large intestine was not altered in mice administered with *L. casei* (Figure 1A, B). In *B. breve*-treated animals, the number of IL-17- and IFN-γ-producing T cells in the small intestine and the large intestine was not significantly changed. However, the number of IL-10-producing T cells was increased about two-fold in the large intestine, but not altered in the small intestine, spleen, and mesenteric lymph nodes (MLN) (Figure 1C, D and Figure S1). Thus, administration of *B. breve* in C57BL/6 mice selectively increased the number of IL-10-producing CD4^+^ T cells in the large intestine without modulating intestinal microbiota.

### *B. breve* induces Foxp3^−^ IL-10-producing T cells

**Figure 2:**
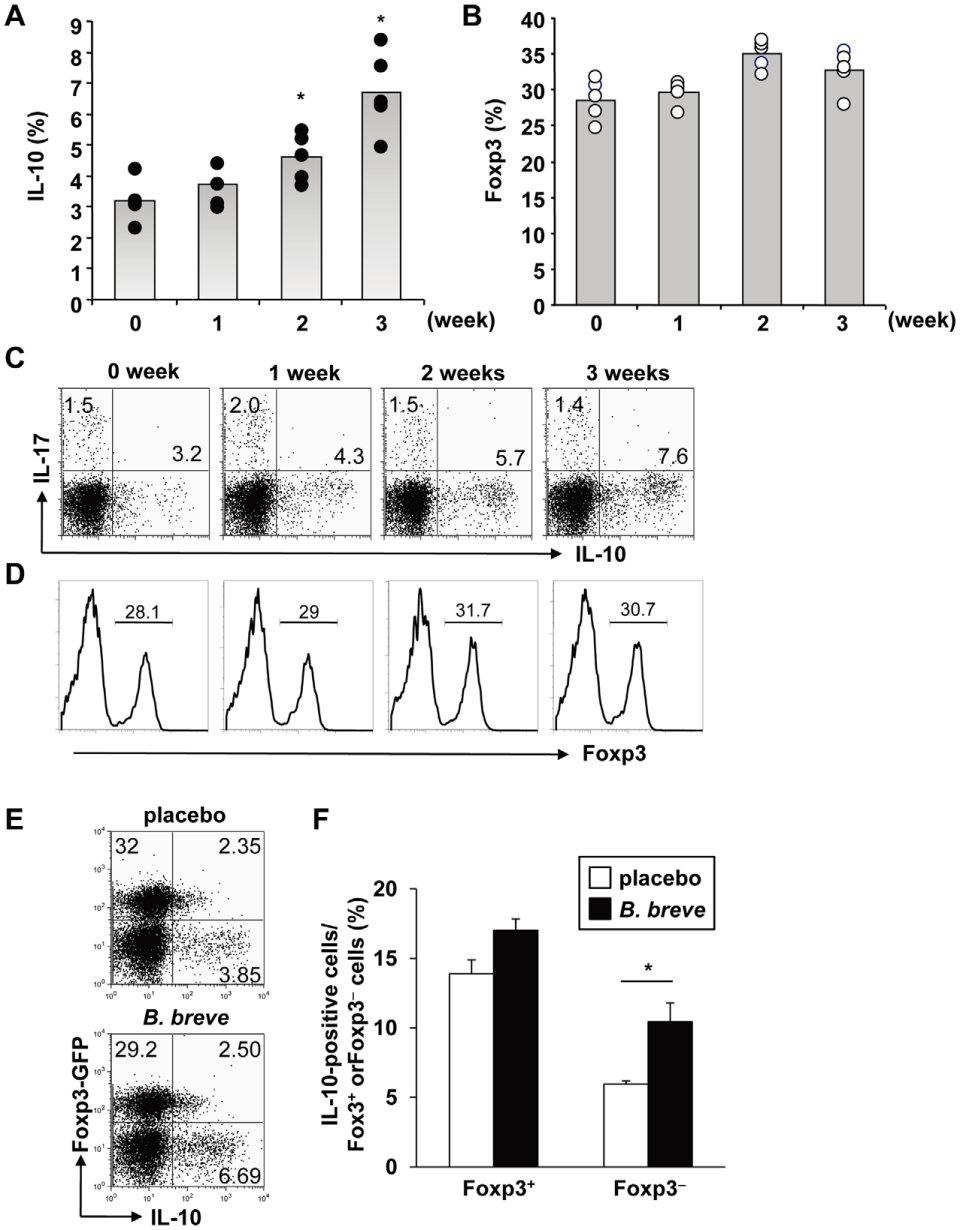
Induction of Foxp3^−^ IL-10-producing CD4^+^ T cells by *B. breve*. 6-week-old BALB/c mice were administered with *B. breve* or placebo orally for 1–4 weeks (n = 5). At the indicated time point, mice were sacrificed and CD4^+^ T cells in the colonic lamina propria were analyzed by flow cytometry. Percentages of CD4^+^ IL-10^+^ T cells (**A**), and CD4^+^ Foxp3^+^ T cells (**B**) are shown. *P<0.02. Representative FACS dot plots for IL-10^+^ and IL-17^+^ T cells (**C**), and histogram for Foxp3^+^ T cells (**D**) gated on CD4^+^ T cells are shown. (**E**) *Foxp3* ^GFP^ mice were fed with *B. breve* for 4 weeks. CD4^+^ T cells in the colonic lamina propria were analyzed for expression of GFP and IL-10. (**F**) Percentages of IL-10^+^ T cells in Foxp3^+^ or Foxp3^−^ CD4^+^ T cells (n = 5). Data are representative of two independent experiments; means ± S.D. *P<0.02.

We next analyzed the effect of *B. breve* on the BALB/c mouse strain. BALB/c mice were orally treated with *B. breve* (10^9^ bacteria) for the indicated time before expression of IL-10 in CD4^+^ T cells of the large intestinal lamina propria was analyzed. The number of colonic IL-10-producing T cells increased after 2 weeks of treatment, and by 3 weeks the number of IL-10-producing cells had doubled (Figure 2A, C). Because IL-10 has been shown to be produced from Foxp3^+^ and Foxp3^−^ populations of intestinal T cells, we analyzed expression of Foxp3 in colonic T cells in *B. breve*-treated BALB/c mice. The number of Foxp3^+^ CD4^+^ T cells in the large intestine was not altered in *B. breve*-treated mice (Figure 2B, D). Therefore, we orally administered *B. breve* into Foxp3-GFP mice, and analyzed IL-10 expression in the colonic CD4^+^ T cells 3 weeks after beginning treatment. The number of IL-10-producing cells was increased in the Foxp3^−^ population, but not in the Foxp3^+^ population of *B. breve*-treated mice (Figure 2 E, F). Thus, *B. breve* administration selectively increased IL-10-producing Foxp3^−^ CD4^+^ T cells in the large intestine.

### Intestinal CD103^+^ DCs promote *B. breve*-dependent Tr1 cell development

**Figure 3:**
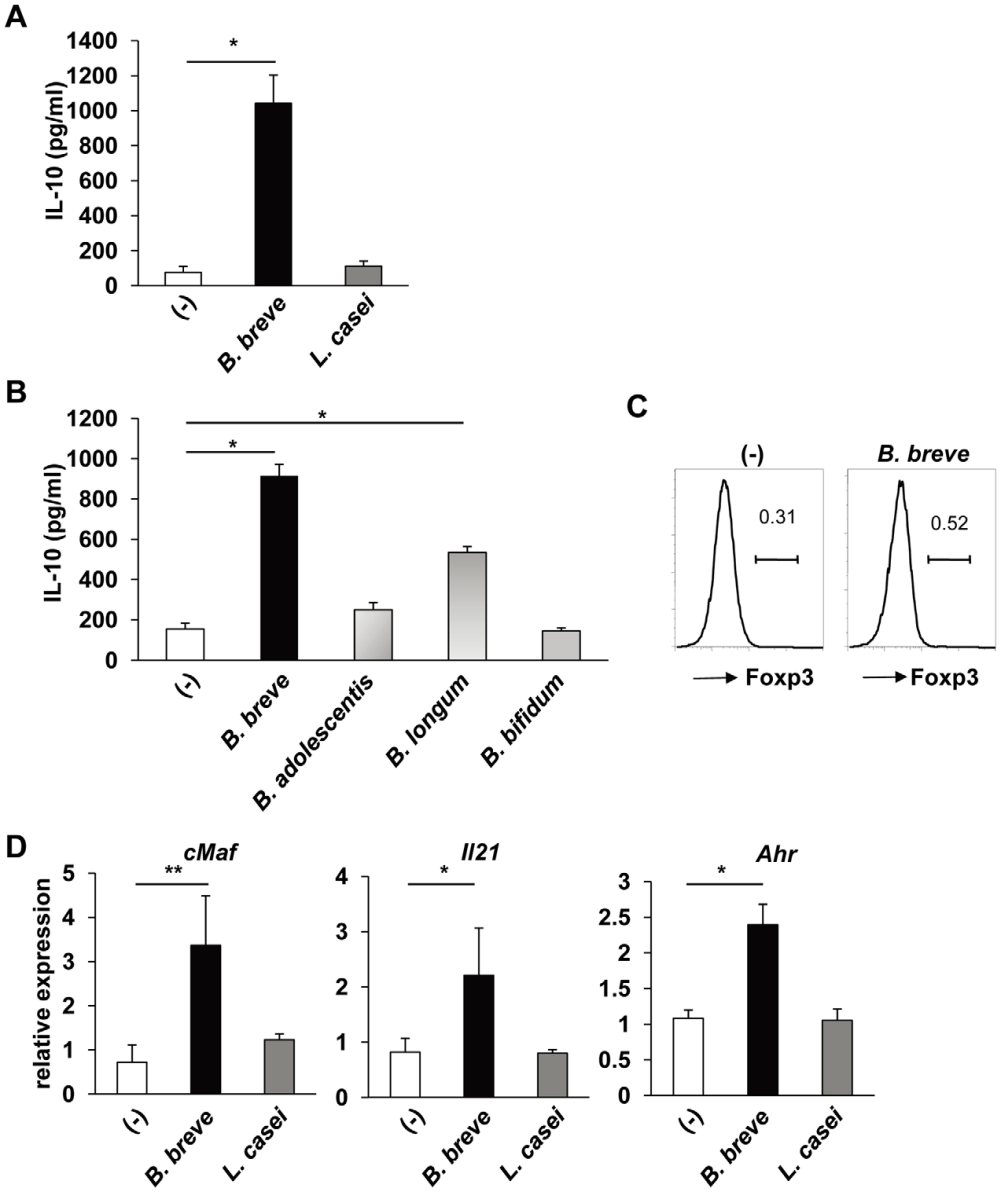
Intestinal DCs mediate *B. breve*-dependent Tr1 cell development. CD11c^+^ DCs (5×10^4^) were isolated from the colonic lamina propria, and cultured with *B. breve*, *L. casei, B. adolescentis, B. longum*, or *B. bifidum* (5×10^4^) for 24 h. After washing, DCs were co-cultured with splenic naïve CD4^+^ T cells (5×10^4^) in the presence of soluble anti-CD3 mAb for 4 days. (**A**) T cells were harvested and re-stimulated with plate-bound anti-CD3 and soluble anti-CD28 mAbs for 24 h. IL-10 concentrations in the culture supernatants were analyzed by ELISA. *P<0.001. (**B**) T cells were harvested and re-stimulated with plate-bound anti-CD3 and soluble anti-CD28 mAbs for 24 h. IL-10 concentrations in the culture supernatants were analyzed by ELISA. *P<0.001. (**C**) T cells were collected, and then stained for CD4 and Foxp3. Foxp3 expression in CD4^+^ cells is shown. (**D**) T cells were harvested, and stimulated with anti-CD3 and anti-CD28 mAbs for 4 h. Total RNA was then extracted to analyze expression of *cMaf*, *Il21*, and *Ahr* by quantitative real-time RT-PCR. Data are representative of five independent experiments and show mean values ± S.D. of triplicate determinations. *P<0.05, **P<0.01.

We next analyzed how *B. breve* induces IL-10-producing T cells. Because intestinal dendritic cells (DCs) modulate T cell differentiation into effector or regulatory T cells, CD11c^+^ cells were isolated from the colonic lamina propria, stimulated with *B. breve* or *L. casei*, and then co-cultured with splenic naïve CD4^+^ T cells. After 4 days of the co-culture, T cells were harvested and stimulated with coated anti-CD3 mAb and soluble anti-CD28 mAb. CD4^+^ T cells co-cultured with *B. breve*-treated, but not *L. casei*-treated, intestinal DCs produced high amounts of IL-10 (Figure 3A). We analyzed the effect of other *Bifidobacterium* spp. Intestinal DCs treated with *B. adolescentis* or *B. bifidum* did not induce IL-10 production from co-cultured CD4^+^ T cells, although *B. longum*-treated DCs moderately induced IL-10-producing T cells (Figure 3B). Thus, *B. breve* strongly induced IL-10-producing T cells via activation of intestinal DCs. In contrast to high induction of IL-10, *B. breve*-treated intestinal CD11c^+^ cells did not induce Foxp3 expression in co-cultured CD4^+^ T cells (Figure 3C). IL-10-producing Foxp3^−^ T cells have been characterized as type 1 regulatory T (Tr1) cells expressing c-Maf, aryl hydrocarbon receptor (Ahr) and IL-21 [42]–[44]. Therefore, we analyzed expression of *cMaf*, *Ahr* and *Il21*. Expression of *cMaf*, *Ahr* and *Il21* was increased in CD4^+^ T cells co-cultured with *B. breve*-treated, but not *L. casei*-treated, intestinal CD11c^+^ cells (Figure 3D). These findings indicate that *B. breve*-treated intestinal DCs promote the induction of IL-10-producing Tr1 cells.

**Figure 4:**
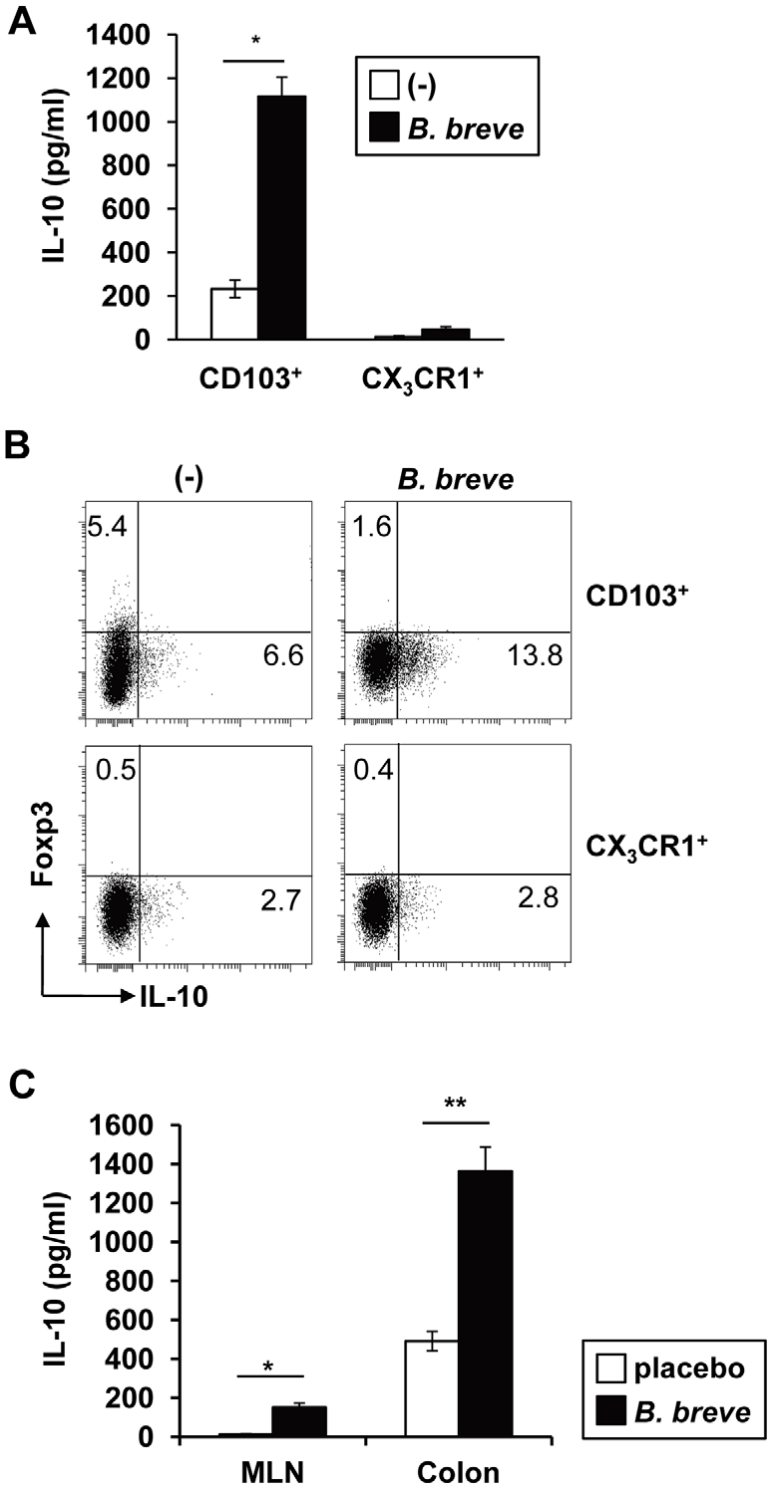
CD103^+^ DCs, but not CX3CR1^+^ DCs, induce *B. breve*-dependent Tr1 cell differentiation. CD103^+^ CX_3_CR1^−^ CD11b^−^ CD11c^+^ DCs (CD103^+^ DCs) and CX_3_CR1^+^ CD11b^+^ CD11c^+^ DCs (CX_3_CR1^+^ DCs) were isolated from the colonic lamina propria, and treated with the same numbers of *B. breve* for 24 h. After washing, splenic naïve CD4^+^ T cells were co-cultured with *B. breve*-treated CD103^+^ DCs or CX_3_CR1^+^ DCs in the presence of anti-CD3 mAb for 4 days. (**A**) T cells were then harvested and re-stimulated for 24 h to analyze IL-10 production by ELISA. *P<0.05. (**B**) T cells were collected, and re-stimulated with PMA and ionophore for 8 h. Intracellular expression of Foxp3 and IL-10 was then analyzed by flow cytometry. (**C**) C57BL/6J mice (n = 5) were fed with *B. breve* for 3 weeks. Then, CD103^+^ DCs were isolated from MLN and the colonic lamina propria, and co-cultured with splenic naïve CD4^+^ T cells. The co-cultured T cells were re-stimulated and IL-10 concentration in the supernatants was analyzed by ELISA. Data are representative of four independent experiments and show mean values ± S.D. of triplicate determinations. *P<0.05, **P<0.01.

Intestinal DCs consists of two major subsets; CD103^+^ CX_3_CR1^−^ CD11b^−^ DCs (CD103^+^ DCs) and CX_3_CR1^+^ CD11b^+^ DCs (CX_3_CR1^+^ DCs) [45], [46]. Therefore, we analyzed which subset mediates *B. breve*-dependent Tr1 cell development. CD103^+^ DCs and CX_3_CR1^+^ DCs were isolated from the colonic lamina propria, treated with *B. breve*, and then co-cultured with naïve CD4^+^ T cells. CD4^+^ T cells co-cultured with *B. breve*-treated CD103^+^ DCs, but not CX_3_CR1^+^ DCs, produced high amounts of IL-10 (Figure 4A, B). *B. breve* caused a dose-dependent increase in IL-10 production from T cells co-cultured with CD103^+^ DCs (Figure S2). CD103^+^ DCs have been shown to induce Foxp3^+^ Treg cells [47], [48]. Indeed, CD103^+^ DCs induced low levels of Foxp3 expression on co-cultured CD4^+^ T cells even in the absence of TGF-β or retinoic acid (Figure 4B). However, *B. breve*-treated CD103^+^ DCs did not induce Foxp3 expression, but induced enhanced IL-10 production in co-cultured T cells.

Next, we analyzed whether intestinal CD103^+^ DCs in *B. breve*-treated mice instruct Tr1 cell development. Since CD103^+^ DCs have been postulated to sample intestinal antigens in the lamina propria and move to MLN where they induce Foxp3^+^ T cells [49], we analyzed CD103^+^ DCs in MLN and colonic lamina propria. C57BL/6 mice were fed with *B. breve* for 3 weeks, before CD103^+^ DCs were isolated from MLN and colonic lamia propria, and co-cultured with naive CD4^+^ T cells. CD4^+^ T cells co-cultured with CD103^+^ DCs from the colonic lamina propria of *B. breve*-fed mice showed higher IL-10 production, with lower levels observed in CD4^+^ T cells co-cultured with MLN CD103^+^ DCs (Figure 4C). Thus, intestinal CD103^+^ DCs possess an enhanced capacity to induce Tr1 cells by *B. breve* treatment in mice. These findings indicate that intestinal CD103^+^ DCs are responsible for *B. breve*-dependent Tr1 cell development.

### *B. breve* induces Tr1 cells via IL-10 and IL-27

**Figure 5:**
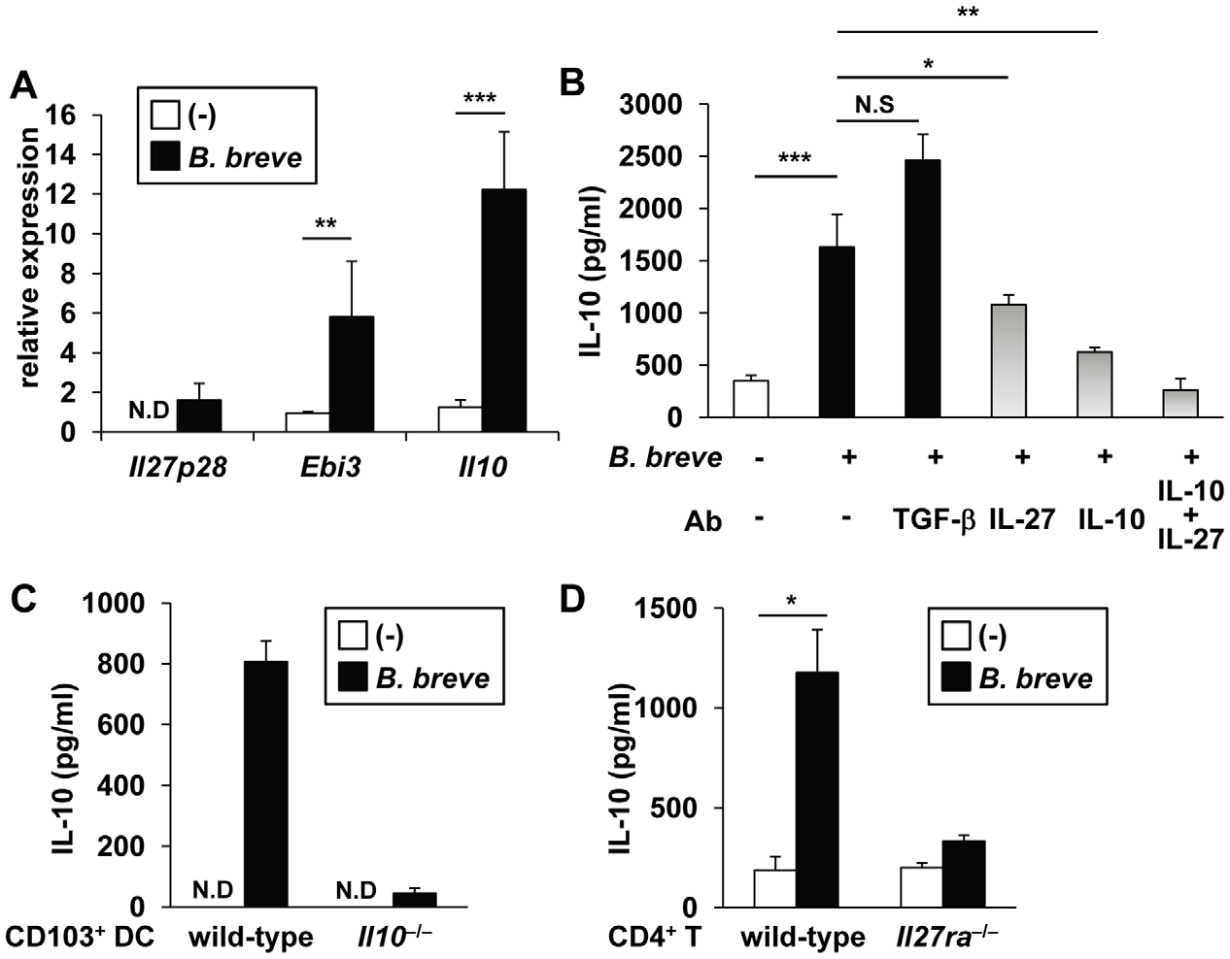
IL-10/IL-27-dependent Tr1 cell development by *B. breve*-treated DCs. (**A**) CD103^+^ DCs were isolated from the colonic lamina propria, and incubated with *B. breve* for 4 h. Total RNA was extracted and analyzed for mRNA expression of *Il27p28*, *Ebi3*, and *Il10* by quantitative real-time RT-PCR. N.D, not detected. **P<0.01, ***P<0.001. (**B**) Naïve T cells were co-cultured with *B. breve*-treated CD103^+^ DC in the presence of the indicated neutralizing antibody for 4 days. T cells were then harvested and re-stimulated with anti-CD3 and CD28 mAbs for 24 h. IL-10 concentrations in the supernatants were measured by ELISA. *P<0.05, **P<0.01, ***P<0.001, N.S, not significant. (**C**) CD103^+^ DCs were isolated from the colonic lamina propria of wild-type and *Il10* ^−/−^ mice (C57BL/6 background) and incubated with *B. breve*. Naïve CD4^+^ T cells from wild-type C57BL/6 mice were then co-cultured with *B. breve*-treated DCs. T cell production of IL-10 was analyzed by ELISA. N.D, not detected. (**D**) CD103^+^ DCs were isolated from the colonic lamina propria of wild-type BALB/c mice and incubated with *B. breve*. Naïve CD4^+^ T cells from wild-type and *Il27ra* ^−/−^ mice (BALB/c background) were then co-cultured with *B. breve*-treated DCs. T cell production of IL-10 was measured by ELISA. Data are representative of three independent experiments and show mean values ± S.D. of triplicate determinations. *P<0.05.

IL-10 was originally shown to induce Tr1 cells [50]. Subsequently, IL-27 was identified as a growth and differentiation factor for Tr1 cells [51]–[53]. Therefore, we analyzed expression of these key cytokines in *B. breve*-treated CD103^+^ DCs. *B. breve* treatment increased expression of *Il27p28*, *Ebi3* (both of which encode subunits of IL-27), and *Il10* in CD103^+^ DCs (Figure 5A). Furthermore, neutralizing mAb to IL-10 or IL-27 severely or moderately blocked *B. breve*-mediated development of Tr1 cells, respectively, and combination of both mAbs almost completely blocked Tr1 cell development. In contrast, neither a retinoic acid receptor antagonist LE540 nor anti-TGF-β neutralizing Ab inhibited *B. breve*-mediated Tr1 cell induction (Figure 5B and Figure S3). These findings indicate that IL-10 and IL-27, which are produced from *B. breve*-treated CD103^+^ DCs, mediate Tr1 cell development. In order to corroborate these findings, we analyzed *Il10* ^−/−^ and *Il27ra* ^−/−^ mice. We first treated CD103^+^ DCs from the colonic lamina propria of wild-type or *Il10* ^−/−^ mice with *B. breve*, before co-culturing them with wild-type naïve CD4^+^ T cells. CD4^+^ T cells co-cultured with *B. breve*-treated *Il10* ^−/−^ DCs produced severely decreased levels of IL-10 (Figure 5C). Then, CD4^+^ T cells were isolated from the spleen of *Il27ra* ^−/−^ mice and co-cultured with *B. breve*-treated wild-type CD103^+^ DCs. IL-10 production from *Il27ra* ^−/−^ T cells was severely decreased (Figure 5D). Taken together, these findings demonstrate that IL-10 and IL-27, which are produced by *B. breve*-treated CD103^+^ DCs, cooperatively mediateTr1 cell induction.

### *B. breve* induces Tr1 cells in a TLR2/MyD88-dependent manner

**Figure 6:**
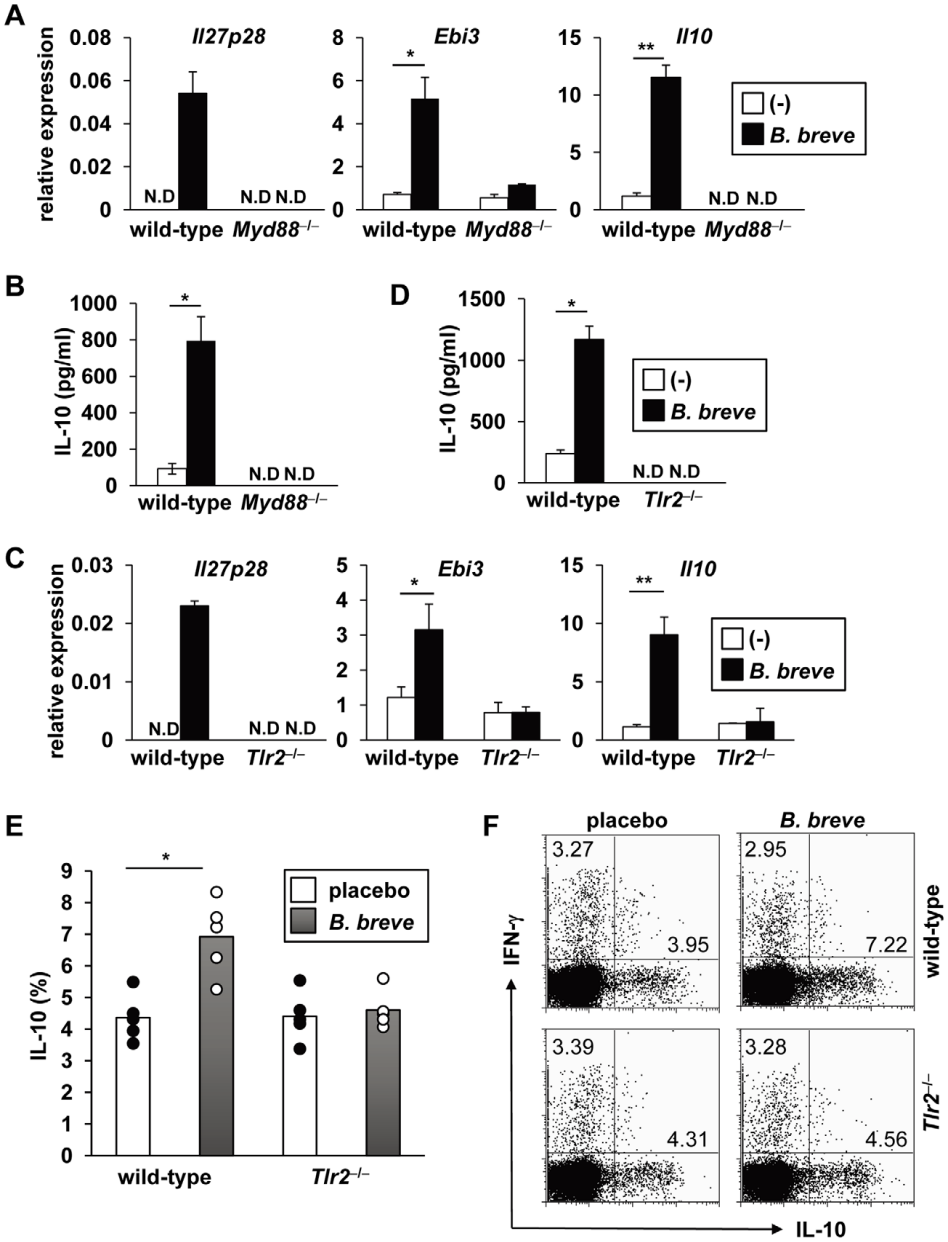
*B. breve* induces Tr1 cells in a TLR2/MyD88-dependent manner. (**A, C**) CD103^+^ DCs were isolated from the colonic lamina propria of wild-type, *Myd88* ^−/−^ (A) and *Tlr2* ^−/−^ (C) mice, incubated with *B. breve* for 4 h, and then analyzed for mRNA expression of *Il27p28*, *Ebi3*, and *Il10*. *P<0.05, **P<0.01. (**B, D**) Wild-type, *Myd88* ^−/−^ (B) and *Tlr2* ^−/−^ (D) CD103^+^ DCs were incubated with *B. breve* for 24 h, and then co-cultured with naïve CD4^+^ T cells from wild-type mice for 4 days. T cells were harvested and re-stimulated for 24 h. IL-10 production in the supernatants was analyzed by ELISA. Data are representative of three independent experiments and show mean values ± S.D. of triplicate determinations. *P<0.05. N.D, not detected. (**E**) 6-week-old wild-type and *Tlr2* ^−/−^ mice (BALB/c background) were fed with *B. breve* or placebo for 3 weeks (n = 5). Then, the mice were sacrificed and colonic lamina propria lymphocytes were analyzed for IL-10 production by flow cytometry. The percentage of IL-10^+^ cells gated on CD4^+^ T cells is shown in the indicated mice. Data are representative of three independent experiments and show mean values ± S.D. of triplicate determinations. *P<0.05. (**F**) Representative FACS plots of IL-10- and IFN-γ-producing CD4^+^ T cells were shown.

We next analyzed which signaling pathway is responsible for *B. breve*-dependent production of IL-10 and IL-27 from CD103^+^ DCs. Several pattern recognition receptors mediate activation of innate immunity through the recognition of microbe-associated molecular patterns [54]. Therefore, we analyzed the involvement of Toll-like receptor (TLR) signaling using *Myd88* ^−/−^ mice. In intestinal CD103^+^ DCs from *Myd88* ^−/−^ mice, *B. breve*-induced expression of *Il27p28*, *Ebi3*, and *Il10* was severely reduced (Figure 6A). Furthermore, wild-type CD4^+^ T cells, which were co-cultured with *B. breve*-treated *Myd88* ^−/−^ CD103^+^ DCs, failed to produce IL-10 (Figure 6B). These findings indicate that the TLR signaling pathway in CD103^+^ DCs is critically involved in *B. breve*-mediated Tr1 cell development. We further analyzed which TLR mediates *B. breve*-mediated responses. *B. breve*-induced expression of *Il27p28*, *Ebi3*, and *Il10* was severely reduced in intestinal CD103^+^ DCs of *Tlr2* ^−/−^ mice (Figure 6C). In addition, *B. breve*-treated *Tlr2* ^−/−^ CD103^+^ DCs did not promote the development of IL-10-producing T cells (Figure 6D). *B. breve*-treated *Tlr4* ^−/−^ and *Tlr9* ^−/−^ CD103^+^ DCs induced IL-10-producing cells normally (Figure S4). CD103^+^ DCs treated with the TLR2 ligand, but not TLR4 or TLR5 ligand, induced Tr1 cells, albeit reduced when compared with *B. breve* (Figure S5). The critical involvement of the TLR2-mediated pathway in *B. breve*-dependent Tr1 induction was further confirmed in *Tlr2* ^−/−^ mice orally administered with *B. breve* for 4 weeks (Figure 6E, F). In *Tlr2* ^−/−^ mice, *B. breve* treatment did not increase the number of IL-10-producing CD4^+^ T cells in the colonic lamina propria. Taken together, these findings demonstrate that the TLR2/MyD88-dependent pathway in CD103^+^ DCs mediates *B. breve*-mediated Tr1 cell induction.

### *B. breve* improves intestinal inflammation via induction of T cell IL-10 production

**Figure 7:**
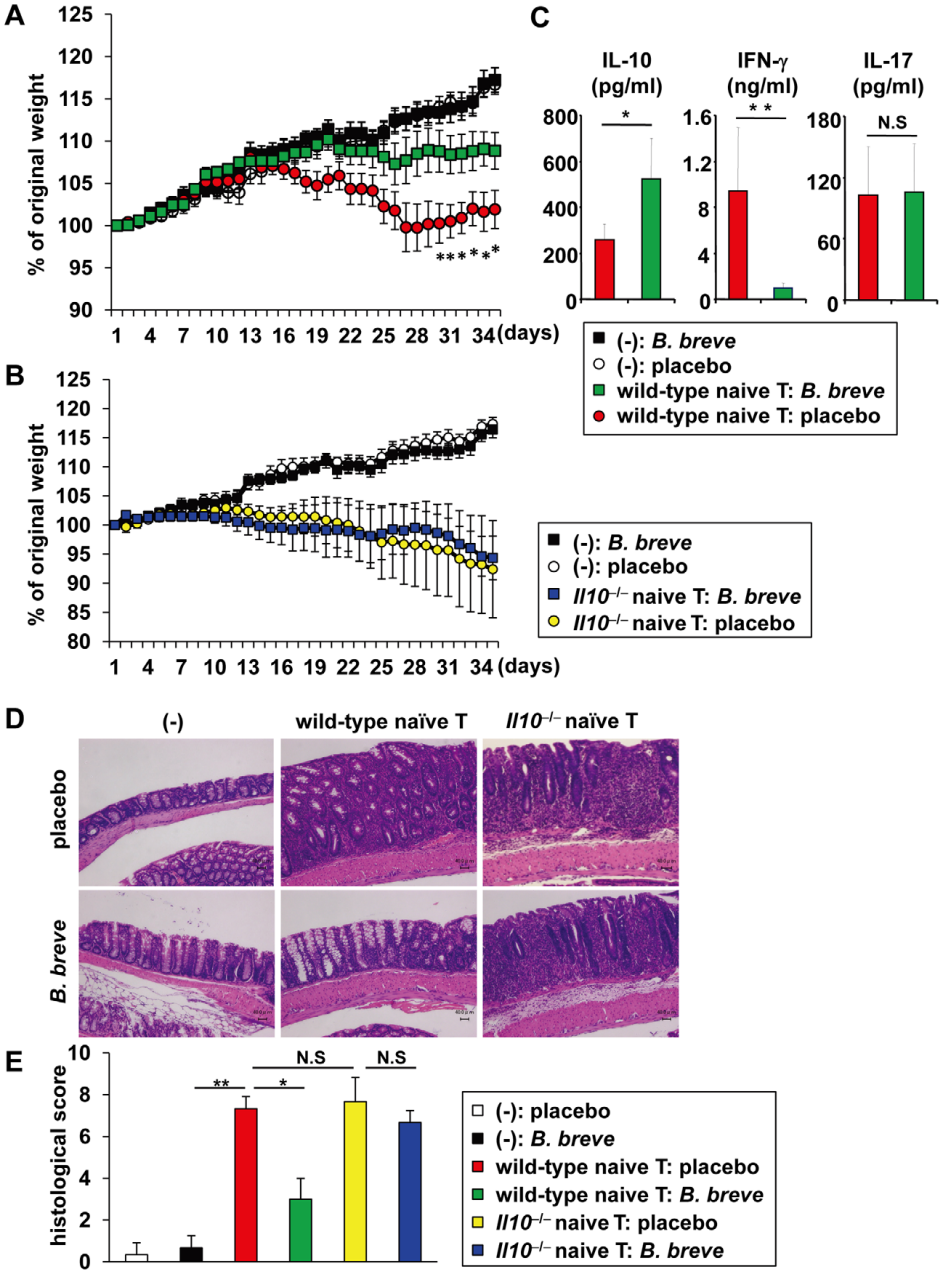
IL-10-dependent amelioration of intestinal inflammation by *B. breve*. (**A, C**) 6 week-old SCID mice (n = 8 per group) were intraperitoneally injected with PBS or 3×10^5^ of naïve CD4^+^ T cells from wild-type BALB/c mice (A) or *Il10* ^−/−^ mice (BALB/c background) (**B**). The mice were orally administered daily with *B. breve* from 1 week before the T cell transfer to the end of experiment. Changes in body weight were monitored daily and presented relative to initial body weight. *P<0.05, Error bars, S.E.M. (**C**) Production of IL-10, IL-17 and IFN-γ from the colon of wild-type T cell-transferred SCID mice daily administered with *B. breve* or placebo (n = 5 per group). *P<0.0064, **P<0.0005. (**D**) Hematoxylin and eosin staining of colon sections at 4 weeks after the transfer. Original magnification, ×400. (**E**) Clinical scores for colitis were shown in the indicated group. Data are representative of two independent experiments. *P<0.05, **P<0.01. N.S, not significant.

Probiotic strains of bacteria have been shown to be used for the treatment of several diseases including IBD [5]–[8]. Therefore, we analyzed the effect of oral *B. breve* treatment in intestinal inflammation caused by transfer of naïve CD4^+^ T cells into immune-compromised severe combined immunodeficiency (SCID) mice. Daily treatment with *B. breve* markedly improved the severity of intestinal inflammation (Figure 7 A, D, E). In *B. breve*-treated SCID mice, IL-10 concentration in the colonic tissues was increased, whereas IFN-γ concentration was decreased (Figure 7C). We then analyzed whether IL-10 was responsible for the prevention of intestinal inflammation. SCID mice were transferred with naïve CD4^+^ T cells from *Il10* ^−/−^ mice and orally treated with *B. breve*. No effect on the amelioration of intestinal inflammation in SCID mice given *Il10* ^−/−^ CD4^+^ T cells was observed (Figure 7B, D, E). These findings demonstrate that T cell-derived IL-10 suppresses T cell-dependent intestinal inflammation in *B. breve*-treated SCID mice.

## Discussion

In the present study, we show that probiotic *B. breve* promotes development of IL-10-producing Tr1 cells in the colon without altering the composition of intestinal commensal flora. Intestinal CD103^+^ DCs mediate *B. breve*-induced development of Tr1 cells via the TLR2/MyD88-dependent induction of IL-27 and IL-10. Recent accumulating evidence has indicated that specific microbiota influence the development of intestinal T cells. Segmented filamentous bacteria have been shown to induce Th17 cells in the small intestine [18], [19]. Polysaccharide A (PSA) of *B. fragilis* has been shown to promote Foxp3^+^ Treg cell development via TLR2 expressed on T cells in the large intestine [21], while *Clostridium* species have been shown to induce Foxp3^+^ Treg cells in the colon through TGF-β induction of epithelial cells [23]. Several probiotic strains of commensal bacteria have also been shown to induce Foxp3^+^ Treg cells or TGF-β expressing Treg cells [27]–[29], [55]. Several studies have also indicated that selective probiotics induce IL-10 production in the intestine or the development of IL-10-producing T cells *in vitro* [26], [29], [56]. However, the precise mechanism by which probiotics induce IL-10-producing T cells in the intestinal lamina propria remained unknown. This study clearly demonstrates that a probiotic strain of bacteria, *B. breve*, promotes development of Foxp3^−^ Tr1-type of T cells.

Several recent studies have demonstrated that colonization of specific microbiota in germ-free mice induced development of Treg cells and Th17 cells [18], [19], [21], [23]. However, oral administration of probiotic *B. breve* did not induce colonic Tr1 cells in germ-free mice. This might be due to that fact that *B. breve* has a low ability to colonize in the intestine by itself. As was the case in other studies [18], [19], [21], [23], germ-free mice received single administration of *B. breve*. However, due to the low ability to colonize in the intestine, *B. breve* might not be able to induce Tr1 cell development by single administration. Alternatively, this probiotic strain might require assistance by other commensal bacteria to be uptaken or recognized by intestinal DCs. A low ability for colonization in the intestine of *B. breve* might correlate with the fact that oral administration of this bacterium did not induce apparent change in the composition of commensal microbiota.

Tr1 cells were identified as the second subset of CD4^+^ regulatory T cells [50]. Both Foxp3^+^ Treg cells and Tr1 cells are critically involved in the maintenance of intestinal homeostasis [30]. *In vitro* studies demonstrated that IL-10 and IL-27 are critical for the induction of Tr1 cells [51]–[53]. The present study shows that intestinal Tr1 cells are induced by both IL-10 and IL-27, which is produced by intestinal CD103^+^ DCs that are exposed to *B. breve*. However, Tr1 cells are present in the intestinal lamina propria of mice that are not fed with *B. breve* [34]. In this regard, given that there are many types of *Bifidobacterium* species in the intestine (Table S1), these indigenous *Bifidobacterium* might contribute to development of intestinal Tr1 cells. Indeed, our data suggest that *B. longum*, one of indigenous commensal bacteria, moderately induced Tr1 cells. The *B. breve*-induced increase in Tr1 cells was observed in the large intestine, but not in the small intestine. This might be due to the characteristics of *B. breve*, which preferentially colonize in the large intestine rather than the small intestine [57].

*B. breve*-induced Tr1 cell development depends on the TLR2/MyD88 pathway. The TLR pathways play mandatory roles in the elimination of pathogenic microorganisms [54]. Previous studies indicated that mice deficient in MyD88, TLR2, or TLR4 were highly sensitive to intestinal inflammation induced by dextran sodium sulfate treatment [58], [59]. However, the mechanism for the TLR-dependent maintenance of gut homeostasis remains unclear. This study demonstrates that the TLR2 pathway in DCs is beneficial for the suppression of intestinal inflammation via induction of IL-10-producing Tr1 cells. It is interesting to note that Tr1 cells are present in *Tlr2* ^−/−^ and *Myd88* ^−/−^ mice, indicating that Tr1 cell development in the intestine in steady states is induced independently of TLR signaling. The TLR-independent induction of intestinal Tr1 cells might be induced by other, so-far unknown, bacteria.

Our *in vitro* experiments clearly indicate that intestinal CD103^+^ CX_3_CR1^−^ CD11b^−^ DCs respond to *B. breve* and promote Tr1 cell development. Intestinal CD103^+^ DCs residing in the colonic lamina propria and MLN showed enhanced capacity to induce IL-10-producing Tr1 cells after *B. breve* treatment. CD103^+^ DCs from MLN were less effective in Tr1 cell induction compared to the lamina proprial CD103^+^ DCs. Thus, it is possible that CD103^+^ DCs in MLN and the colonic lamina propria have differential characteristics in Tr1 cell induction. In addition, it remains unclear how CD103^+^ DCs sense *B. breve* in the intestinal mucosa. CX_3_CR1-expressing intestinal DCs have been shown to extend their dendrites into the intestinal lumen to sample luminal contents [60]. However, CD103^+^ DCs do not express the CX_3_CR1 that is required for dendrite extension. Several metabolites produced by commensal microbiota have been shown to influence host cell gene expression [61]. However, culture supernatants of *B. breve* did not induce IL-10 production from T cells co-cultured with CD103^+^ DCs, indicating that *B. breve* directly acts on intestinal DCs (Figure S6). Elucidating how CD103^+^ DCs recognize *B. breve* in the intestinal lamina propria would be a future interesting issue.

IL-10-producing Tr1 cells can be induced by UV-irradiated *B. breve*, or even sonicated *B. breve* (Figure S7). These findings indicate that components of *B. breve* directly act on intestinal DCs, possibly by interacting with TLR2, and promote Tr1 cell development. TLR2 has been shown to recognize a unique polysaccharide structure (PSA) of *B. fragilis* to induce Foxp3^+^ Treg cells [21]. The probiotic strain of *B. breve* used in this study also possesses a unique structure of polysaccharide in their cell walls [62]. Therefore, it would be interesting in the future to analyze whether the polysaccharide of *B. breve* is recognized by TLR2 to induce Tr1 cells. Identification of such *B. breve* components that activate the TLR2 pathway will lead to development of a new effective agent for the treatment of IBD.

In contrast to the development of Tr1 cells promoted by *B. breve*, *L. casei* did not have any effect on the differentiation of intestinal T cells, although it is well known as a beneficial probiotic strain possessing several health-promoting effects [63]. In this regard, several mechanisms of action of probiotics, other than the influence on the host T cell development, have been postulated [7], [8]. These include enhancement of barrier functions of epithelial cells, modification of commensal flora, and effects on dendritic cells and monocytes/macrophages. Several mechanisms of *Lactobaciluus* species-mediated actions have been reported [55], [56], [64]. Our results indicate that each probiotic strain has their specific modes of action on the host. VSL#3 containing several probiotics (three bifidobacteria, five lactobacilli and *Streptococcus salivarius* subsp. *thermophilus*) have been reported to have potent effects on host health and diseases [26]. This might be due to the synergistic effect of these different probiotic strains that have distinct mechanisms of actions.

In the present study, we show that a probiotic bacterium, *B. breve*, induces intestinal Tr1 cells and thereby improves intestinal inflammation. Analysis of the effect of this probiotic-dependent Tr1 cell development on other disease models will expand the application of *B. breve* as a therapeutic agent.

## Materials and Methods

### Ethics statement

All animal experiments were carried out in strict accordance with the Guidelines for Animal Experimentation of the Japanese Association for Laboratory Animal Science. The protocol was approved by the committee for Animal Experiments of Osaka University (Permit Number: 21-058-0).

### Reagents

*Lactobacilus casei* strain Shirota (*L. casei*) and *Bifidobacterium breve* Yakult strain (*B. breve*) were as described [35], [40]. *B. bifidum* (Yakult strain YIT10347), *B. adolescntis* (ATCC15703), and *B. longum* (ATCC15707) were used for experiments. For oral treatment of mice, freeze-dried preparations of *L. casei* and *B. breve* were dissolved with distilled water, and 1×10^9^ bacteria were administered. A sachet of *B. breve* contained 4×10^9^ freeze-dried living bacteria, cornstarch, and hydroxypropyl cellulose as vehicle. Placebo sachet of *B. breve* contained only cornstarch and hydroxypropyl cellulose. A sachet of *L. casei* consisted of 5×10^9^ freeze-dried living bacteria with lactose, cornstarch, powdered skim milk, crystallized cellulose and hydroxypropyl cellulose. The placebo sachet of *L. casei* consisted of only common excipients. For *in vitro* stimulation, *B. breve* was inoculated in GAM broth (Nissui Pharmaceutical) supplemented with 1%(w/v) glucose, and cultured for 24 h at 37°C under anaerobic conditions, and then centrifuged and the pellets were suspended with culture media. The number of *B. breve* was measured by culturing on MRS agar plate. Neutralizing anti-mouse IL-10 was purchased from BD biosciences, anti-mouse IL-27p28, and anti-TGF-β (1D11) blocking antibodies were purchased from R&D systems. Anti-mouse CD3 (145-2C11) and CD28 (37.51) were obtained from BioLegend. LE540 was purchased from WAKO Chemicals (Tokyo, Japan).

### Animals

BALB/c and C57BL/6J mice were purchased from CLEA Japan or Japan SLC. CB17-SCID mice were obtained from CLEA Japan. *Il10* ^−/−^, *Foxp3* ^eGFP^ were purchased from Jackson laboratories, and *Myd88* ^−/−^, *Tlr2* ^−/−^, *Tlr4* ^−/−^ and *Tlr9* ^−/−^ mice were generated previously [65]. *Il27ra* ^−/−^ mice were kindly provided by Amgen [66]. These mice were backcrossed eight or more generations onto BALB/c or C57BL/6J. C57BL/6J mice were orally administered with *L. casei* or *B. breve* (10^9^ bacteria each) as well as placebo daily with gastric tubes for 3 months. Alternatively, probiotics were orally introduced into BALB/c, C57BL/6J, or *Foxp3* ^eGFP^ mice for 1–4 weeks.

### Analysis of fecal microbiota

Methods for the analysis of fecal bacteria are described in Text S1.

### Flow cytometry

For flow cytometry, the following antibodies were used: PerCP/Cy5.5-conjugated anti-CD4 (GK1.5), Alexa Fluor 647-conjugated CD11c (N418), CD62L (MEL-14), streptavidin-conjugated PE/Cy7 from BioLegend, FITC-conjugated anti-CD11b (M1/70), CD25 (7D4), and PE-conjugated CD103, anti-mouse CD16/32 (Fcγ III/II receptor) from BD PharMingen, PE-conjugated CD44 (IM7), Alexa Fluor 647-conjugated Foxp3 (FJK-16s), and biotin-conjugated CX3CR1 from eBiosciences. Flow cytometric analysis was performed using a FACS Canto II flow cytometer (BD Biosciences) with FlowJo software (Tree Star).

### Isolation of intestinal lamina propria DC subsets and lymphocytes

Lamina propria DCs and lymphocytes were isolated as previously described [67] with simple modifications. Briefly, colons and small intestines were opened longitudinally and vigorously rinsed in PBS. Intestines were shaken in HBSS containing 5 mM EDTA and 5% fetal bovine serum (FBS) for 20 min at 37°C. After removal of epithelial layers and fat tissues, the intestines were cut into small pieces and incubated with RPMI 1640 containing 5% FBS, 1 mg/ml of collagenase D (Roche Diagnostics), 1 mg/ml of dispase (Invitrogen) and 40 µg/ml of DNase I (Roche Diagnostics) for 1 h at 37°C in a shaking water bath. The digested tissues were washed with HBSS containing 5 mM EDTA. Cell suspensions were filtered through a 40 µm cell strainer into chilled PBS and centrifuged. Cell suspensions from enzyme digestion were then applied to a Percoll (GE Healthcare) gradient (for DCs: 30% percoll on top, 75% percoll on the bottom, and for lymphocytes: 40% percoll on top, 80% percoll on the bottom) by centrifugation at 780 *g* for 20 min at 25°C. The cells at interface were taken and washed twice with FACS buffer. For purifying lamina propria DC subsets, single cell suspensions were treated with anti-mouse Fcγ receptor antibody for 5 min at 4°C. Cells were then stained with CD11c-APC, CD11b-FITC, CD103-PE and CX3CR1-PE-Cy7 and subsequently sorted using a FACSAria (BD Biosciences) to a purity >98%. The cells were used immediately for each of experiment.

### Isolation of splenic naïve CD4^+^ cells

To prepare single-cell suspensions from spleens, they were ground between glass slides and passed through a 40 µm cell strainer. Splenocytes were treated with RBC lysis buffer (0.15 M NH_4_Cl, 1 mM KHCO_3_, 0.1 mM EDTA) for 5 min and washed twice with PBS. For FACS sorting, cells were stained with PerCP/Cy5.5-conjugated anti-CD4 (Biolegend), APC-conjugated anti-CD62L, FITC-conjugated anti-CD25 and PE-conjugated anti-CD44 (BD Biosciences). Naïve CD4^+^ T cells were sorted using a FACSAria for CD4^+^CD62L^high^CD25^−^CD44^low^. The purity of the sorted cells was routinely >98%.

### Intracellular cytokine staining

The intracellular expression of IFN-γ, IL-17, and IL-10 in CD4^+^ T cells was analyzed using the Cytofix/Cytoperm Kit Plus (with Golgistop; BD Biosciences) according to the manufacturer's instructions. In brief, lymphocytes obtained from the intestinal lamina propria were incubated with 50 ng/ml of phorbol myristate acetate (PMA; Sigma) and 5 µM of calcium ionophore A23187 (Sigma) and Golgistop in complete RPMI1640 at 37°C for 4 h. Surface staining was performed with PerCP/Cy5.5-conjufated anti-CD4 for 20 min at 4°C. After Fix/Perm treatment for 20 min, intracellular cytokine staining was performed with PE-conjugated anti-IL-10, FITC-conjugated anti-IFN-γ, and APC-conjugated anti-IL-17 for 20 min. Data were acquired using a FACS Canto II and analyzed using FlowJo software. Alternatively, for intracellular staining for Foxp3 and IL-10, cells were stained using the Foxp3 Staining Buffer set (eBiosciences).

### *In vitro* co-culture assays

Colonic DC subsets (5×10^4^) were incubated with the same number or the indicated number of *L. casei* or *B. breve* in 100 µl of complete RPMI1640 media for 24 h in a round-bottom 96 well plate. DCs were then washed with PBS and naïve CD4^+^ T cells (5×10^4^) were added into the culture with 2 µg/ml soluble anti-CD3 mAb. After 4 days, T cells were collected, washed and counted. The same numbers of T cells were re-stimulated with plate-bound anti-CD3 mAb (2 µg/ml) and soluble anti-CD28 mAb (2 µg/ml) for 24 h. Re-stimulated T cell cytokine production in the supernatants was analyzed by ELISA (R&D systems). Alternatively, T cells were re-stimulated with 50 ng/ml of PMA and 5 µM of calcium ionophore A23187 for 6 h before intracellular cytokine staining was performed as described above. Golgistop was added for the last 2 h.

### Quantitative real-time RT–PCR

Total RNA was isolated with the RNeasy Mini Kit (Qiagen), and 1–2 µg of total RNA was reverse transcribed using M-MLV reverse transcriptase (Promega) and random primers (Toyobo) after treatment with RQ1 DNase I (Promega). Complementary DNAs were analyzed by qPCR using the GoTaq qPCR Master Mix (Promega) on an ABI 7300 system (Applied Biosystems). All values were normalized to the expression of *Gapdh* encoding glyceraldhyde-3-phosphate dehydrogenase, and the fold difference in expression relative to that for *Gapdh* is shown. Amplification conditions were: 50°C (2 min), 95°C (10 min), and 40 cycles of 95°C (15 s) and 60°C (60 s). The following primer sets were used: *cMaf*, 5′-AATCCTGGCCTGTTTCACAT-3′ and 5′-TGACGCCAACATAGGAGGTG-3′; *Il21*, 5′-GCCAGATCGCCTCCTGATTA-3′ and 5′-CATGCTCACAGTGCCCCTTT-3′; *Il27p28*, 5′-TTCCCAATGTTTCCCTGACTTT-3′ and 5′-AAGTGTGGTAGCGAGGAAGCA-3′; *Ebi3*, 5′-TGAAACAGCTCTCGTGGCTCTA-3′ and 5′-GCCACGGGATACCGAGAA-3′; *Il10*, 5′-TTTCAAACAAAGGACCAG-3′ and 5′-GGATCATTTCCGATAAGG-3′; and *Gapdh*, 5′-TGTGTCCGTCGTGGATCTGA-3′ and 5′-CCTGCTTCACCACCTTCTTGA-3′

### T-cell-mediated colitis model

Naive CD4^+^CD62L^high^CD25^−^CD44^low^ splenic T cells from BALB/c mice or *Il10* ^−/−^ mice (BALB/c background) were purified and intraperitoneally transferred into SCID mice (3×10^5^ cells per mouse). *B. breve* (10^9^ bacteria) were fed by oral gavage from 3 days before the transfer to the end of the experiments. Weight changes were monitored every day. The mice were sacrificed, and the colons were examined histochemically after haematoxylin and eosin staining. Alternatively, the colons were cut into small pieces after wash and cultured for 24 h. Then, culture supernatants were collected and the level of IL-10, IL-17A and IFN-γ was measured by ELISA (R&D systems).

### Histopathological analysis

Paraffin-embedded colon samples were sectioned and stained with hematoxylin and eosin. Severity of colitis was evaluated by the standard scoring system as previously described [68]. Five regions of the colon (cecum; ascending, transverse, and descending of colon; and rectum) were graded semiquantitatively from 0 (no change) to 5 (most severe change). The grading represents an increasing incidence and degree of inflammation, goblet cell loss, ulceration and fibrosis in the lamina propria. The scoring was performed in a blinded manner. Images of hematoxylin and eosin staining and May-Grunwald-Giemsa staining were taken using Biozero (Keyence).

### Statistical analysis

Statistical analysis was performed using PRISM 4 software. Unpaired student's *t*-test and Mann-Whitney U test were used to determine the significance of experiments. P values of less than 0.05 were considered statistically significant.

## Supporting Information

Figure S1 **Percentage of IL-10^+^ or Foxp3^+^ CD4^+^ T cells in MLN or spleens were not changed by oral treatment of** ***B. breve*** **.** 6-week-old C57BL/6 mice were fed with *B. breve* or placebo daily by oral gavage for 3 months (n = 8). MLNs and spleens were taken, and analyzed for expression of cytokines and Foxp3 by flow cytometry. Representative FACS dot plots were shown gated on CD4^+^ T cells. **A**: MLN, **B**: Spleen.
(PDF)

Figure S2 ***B. breve*** **induces IL-10-producing Tr1 cells in a dose-dependent manner.** CD11c^high^ CD11b^−^CD103^+^ DCs (CD103^+^ DCs) (5×10^4^) were isolated from the colonic lamina propria of C57BL/6J mice, and treated with the increasing numbers of *B. breve* (5×10^1^ to 5×10^5^) for 24 h in round-bottom 96-well plate. After washing, splenic naïve CD4^+^ T cells (5×10^4^) were co-cultured with *B.breve*-treated CD103^+^ DC in the presence of anti-CD3 mAb for 4 days. Then, T cells were harvested and re-stimulated. IL-10 production in the culture supernatants was analyzed by ELISA. Data are representative of two independent experiments. Error bars, S.D. *P<0.05, **P<0.01.
(PDF)

Figure S3 **Retinoic acid-independent induction of Tr1 cells by** ***B. breve*** **.** *B. breve*-treated CD103^+^ DCs were co-cultured with splenic naïve CD4^+^ T cells in the presence of an inhibitor of retinoic acid receptor (2 µM of LE540, WAKO chemicals, JAPAN) for 4 days. IL-10 production by re-stimulated T cells was quantified by ELISA. Data are representative of two independent experiments. Error bars, S.D. *P<0.01, N.S, not significant.
(PDF)

Figure S4 **TLR4/TLR9-independent induction of Tr1 cells by** ***B. breve*** **.** Intestinal CD103^+^ DCs from wild-type, *Tlr4* ^−/−^ and *Tlr9* ^−/−^ mice were treated with *B. breve* for 24 h, and then co-cultured with splenic naïve CD4^+^ T cells for 4 days. IL-10 production by re-stimulated T cells was quantified by ELISA. Data are representative of two independent experiments. Error bars, S.D. *P<0.01.
(PDF)

Figure S5 **TLR2-dependent induction of Tr1 cells.** Intestinal CD103^+^ DCs were stimulated with *B. breve* or TLR ligands such as LPS (TLR4 ligand), Pam3 (TLR2 ligand) or flagellin (TLR5 ligand) for 24 h, and then co-cultured with splenic naïve CD4^+^ T cells for 4 days. IL-10 production by re-stimulated T cells was quantified by ELISA. Data are representative of two independent experiments. Error bars, S.D. *P<0.01.
(PDF)

Figure S6 ***B. breve*** **directly acts on CD103^+^ DCs to induce Tr1 cells.** CD103^+^ DCs were treated by *B.breve* or culture supernatant (10-fold concentrated) of *B. breve* for 24 h. After washing, naïve CD4^+^ T cells were co-cultured with treated CD103^+^ DCs for 4 days. Then, T cells were harvested and re-stimulated by anti-CD3 and anti-CD28 mAbs. IL-10 concentration in the supernatants was quantified by ELISA. Representative data were shown from two independent experiments. Error bars, S.D. N.D, not detected.
(PDF)

Figure S7 **Induction of Tr1 cell development by killed** ***B. breve*** **.** CD103^+^ DCs were treated by live, UV killed or sonicated *B. breve* for 24 h, and then, co-cultured with naïve CD4^+^ T cells for 4 days. T cells were harvested and re-stimulated by anti-CD3 and anti-CD28 mAbs. IL-10 concentration in the supernatants was quantified by ELISA. Data were representative of three independent experiments. Error bars, S.D. *P<0.01, N.S, not significant.
(PDF)

Table S1 **Composition of fecal commensal microflora in probiotics-fed mice.** 6-week-old C57BL/6 mice were fed with *L. casei*, *B. breve* or placebo daily (1×10^9^) by oral gavage for 3 months (n = 5, respectively). Fecal samples were collected, weighed and suspended in 9 volumes of sterilized anaerobic transfer medium. Total RNA and DNA fractions extracted from each sample were assessed by RT-qPCR or qPCR with the specific primers. “Number” indicates CFU of each bacteria calculated using control cultured bacteria. (x/5) indicated the right side of “number” show detection rate of mice analyzed.
(PDF)

Text S1 **Supplemental methods.** Methods for “Analysis of Fecal Microbiota” and “Culture and Killing of *B. breve*” are described with references.
(PDF)

## References

[1] Frank DN, St Amand AL, Feldman RA, Boedeker EC, Harpaz N (2007). Molecular-phylogenetic characterization of microbial community imbalances in human inflammatory bowel diseases.. Proc Natl Acad Sci U S A.

[2] Peterson DA, Frank DN, Pace NR, Gordon JI (2008). Metagenomic approaches for defining the pathogenesis of inflammatory bowel diseases.. Cell Host Microbe.

[3] Qin J, Li R, Raes J, Arumugam M, Burgdorf KS (2010). A human gut microbial gene catalogue established by metagenomic sequencing.. Nature.

[4] Elinav E, Strowig T, Kau AL, Henao-Mejia J, Thaiss CA (2011). NLRP6 inflammasome regulates colonic microbial ecology and risk for colitis.. Cell.

[5] Hart AL, Stagg AJ, Kamm MA (2003). Use of probiotics in the treatment of inflammatory bowel disease.. J Clin Gastroenterol.

[6] Sartor RB (2005). Probiotic therapy of intestinal inflammation and infections.. Curr Opin Gastroenterol.

[7] Boirivant M, Strober W (2007). The mechanism of action of probiotics.. Curr Opin Gastroenterol.

[8] Ng SC, Hart AL, Kamm MA, Stagg AJ, Knight SC (2009). Mechanisms of action of probiotics: recent advances.. Inflamm Bowel Dis.

[9] Martin FP, Wang Y, Sprenger N, Yap IK, Lundstedt T (2008). Probiotic modulation of symbiotic gut microbial-host metabolic interactions in a humanized microbiome mouse model.. Mol Syst Biol.

[10] Sonnenburg JL, Chen CT, Gordon JI (2006). Genomic and metabolic studies of the impact of probiotics on a model gut symbiont and host.. PLoS Biol.

[11] Mennigen R, Bruewer M (2009). Effect of probiotics on intestinal barrier function.. Ann N Y Acad Sci.

[12] Round JL, Mazmanian SK (2009). The gut microbiota shapes intestinal immune responses during health and disease.. Nat Rev Immunol.

[13] Chung H, Kasper DL (2010). Microbiota-stimulated immune mechanisms to maintain gut homeostasis.. Curr Opin Immunol.

[14] Hooper LV, Macpherson AJ (2010). Immune adaptations that maintain homeostasis with the intestinal microbiota.. Nat Rev Immunol.

[15] Hapfelmeier S, Lawson MA, Slack E, Kirundi JK, Stoel M (2010). Reversible microbial colonization of germ-free mice reveals the dynamics of IgA immune responses.. Science.

[16] Slack E, Hapfelmeier S, Stecher B, Velykoredko Y, Stoel M (2009). Innate and adaptive immunity cooperate flexibly to maintain host-microbiota mutualism.. Science.

[17] Lee YK, Mazmanian SK (2010). Has the microbiota played a critical role in the evolution of the adaptive immune system?. Science.

[18] Gaboriau-Routhiau V, Rakotobe S, Lecuyer E, Mulder I, Lan A (2009). The key role of segmented filamentous bacteria in the coordinated maturation of gut helper T cell responses.. Immunity.

[19] Ivanov II, Atarashi K, Manel N, Brodie EL, Shima T (2009). Induction of intestinal Th17 cells by segmented filamentous bacteria.. Cell.

[20] Mazmanian SK, Round JL, Kasper DL (2008). A microbial symbiosis factor prevents intestinal inflammatory disease.. Nature.

[21] Round JL, Mazmanian SK (2010). Inducible Foxp3+ regulatory T-cell development by a commensal bacterium of the intestinal microbiota.. Proc Natl Acad Sci U S A.

[22] Round JL, Lee SM, Li J, Tran G, Jabri B (2011). The Toll-like receptor 2 pathway establishes colonization by a commensal of the human microbiota.. Science.

[23] Atarashi K, Tanoue T, Shima T, Imaoka A, Kuwahara T (2011). Induction of colonic regulatory T cells by indigenous Clostridium species.. Science.

[24] Geuking MB, Cahenzli J, Lawson MA, Ng DC, Slack E (2011). Intestinal Bacterial Colonization Induces Mutualistic Regulatory T Cell Responses.. Immunity.

[25] Lathrop SK, Bloom SM, Rao SM, Nutsch K, Lio CW (2011). Peripheral education of the immune system by colonic commensal microbiota.. Nature.

[26] Di Giacinto C, Marinaro M, Sanchez M, Strober W, Boirivant M (2005). Probiotics ameliorate recurrent Th1-mediated murine colitis by inducing IL-10 and IL-10-dependent TGF-beta-bearing regulatory cells.. J Immunol.

[27] Lyons A, O'Mahony D, O'Brien F, MacSharry J, Sheil B (2010). Bacterial strain-specific induction of Foxp3+ T regulatory cells is protective in murine allergy models.. Clin Exp Allergy.

[28] O'Mahony C, Scully P, O'Mahony D, Murphy S, O'Brien F (2008). Commensal-induced regulatory T cells mediate protection against pathogen-stimulated NF-kappaB activation.. PLoS Pathog.

[29] Lavasani S, Dzhambazov B, Nouri M, Fak F, Buske S (2010). A novel probiotic mixture exerts a therapeutic effect on experimental autoimmune encephalomyelitis mediated by IL-10 producing regulatory T cells.. PLoS One.

[30] Barnes MJ, Powrie F (2009). Regulatory T cells reinforce intestinal homeostasis.. Immunity.

[31] Vieira PL, Christensen JR, Minaee S, O'Neill EJ, Barrat FJ (2004). IL-10-secreting regulatory T cells do not express Foxp3 but have comparable regulatory function to naturally occurring CD4+CD25+ regulatory T cells.. J Immunol.

[32] O'Garra A, Vieira PL, Vieira P, Goldfeld AE (2004). IL-10-producing and naturally occurring CD4+ Tregs: limiting collateral damage.. J Clin Invest.

[33] Pot C, Apetoh L, Awasthi A, Kuchroo VK (2010). Molecular pathways in the induction of interleukin-27-driven regulatory type 1 cells.. J Interferon Cytokine Res.

[34] Maynard CL, Harrington LE, Janowski KM, Oliver JR, Zindl CL (2007). Regulatory T cells expressing interleukin 10 develop from Foxp3+ and Foxp3- precursor cells in the absence of interleukin 10.. Nat Immunol.

[35] Matsuzaki T, Nagata Y, Kado S, Uchida K, Kato I (1997). Prevention of onset in an insulin-dependent diabetes mellitus model, NOD mice, by oral feeding of Lactobacillus casei.. APMIS.

[36] Matsuzaki T, Yamazaki R, Hashimoto S, Yokokura T (1997). Antidiabetic effects of an oral administration of Lactobacillus casei in a non-insulin-dependent diabetes mellitus (NIDDM) model using KK-Ay mice.. Endocr J.

[37] Kato I, Endo-Tanaka K, Yokokura T (1998). Suppressive effects of the oral administration of Lactobacillus casei on type II collagen-induced arthritis in DBA/1 mice.. Life Sci.

[38] Matsumoto S, Hara T, Hori T, Mitsuyama K, Nagaoka M (2005). Probiotic Lactobacillus-induced improvement in murine chronic inflammatory bowel disease is associated with the down-regulation of pro-inflammatory cytokines in lamina propria mononuclear cells.. Clin Exp Immunol.

[39] Ishikawa H, Akedo I, Umesaki Y, Tanaka R, Imaoka A (2003). Randomized controlled trial of the effect of bifidobacteria-fermented milk on ulcerative colitis.. J Am Coll Nutr.

[40] Kato K, Mizuno S, Umesaki Y, Ishii Y, Sugitani M (2004). Randomized placebo-controlled trial assessing the effect of bifidobacteria-fermented milk on active ulcerative colitis.. Aliment Pharmacol Ther.

[41] Matsuda K, Tsuji H, Asahara T, Matsumoto K, Takada T (2009). Establishment of an analytical system for the human fecal microbiota, based on reverse transcription-quantitative PCR targeting of multicopy rRNA molecules.. Appl Environ Microbiol.

[42] Roncarolo MG, Gregori S, Battaglia M, Bacchetta R, Fleischhauer K (2006). Interleukin-10-secreting type 1 regulatory T cells in rodents and humans.. Immunol Rev.

[43] Pot C, Jin H, Awasthi A, Liu SM, Lai CY (2009). Cutting edge: IL-27 induces the transcription factor c-Maf, cytokine IL-21, and the costimulatory receptor ICOS that coordinately act together to promote differentiation of IL-10-producing Tr1 cells.. J Immunol.

[44] Apetoh L, Quintana FJ, Pot C, Joller N, Xiao S (2010). The aryl hydrocarbon receptor interacts with c-Maf to promote the differentiation of type 1 regulatory T cells induced by IL-27.. Nat Immunol.

[45] Laffont S, Powrie F (2009). Immunology: Dendritic-cell genealogy.. Nature.

[46] Varol C, Vallon-Eberhard A, Elinav E, Aychek T, Shapira Y (2009). Intestinal lamina propria dendritic cell subsets have different origin and functions.. Immunity.

[47] Sun CM, Hall JA, Blank RB, Bouladoux N, Oukka M (2007). Small intestine lamina propria dendritic cells promote de novo generation of Foxp3 T reg cells via retinoic acid.. J Exp Med.

[48] Coombes JL, Siddiqui KR, Arancibia-Carcamo CV, Hall J, Sun CM (2007). A functionally specialized population of mucosal CD103+ DCs induces Foxp3+ regulatory T cells via a TGF-beta and retinoic acid-dependent mechanism.. J Exp Med.

[49] Scott CL, Aumeunier AM, Mowat AM (2011). Intestinal CD103(+) dendritic cells: master regulators of tolerance?. Trends Immunol.

[50] Groux H, O'Garra A, Bigler M, Rouleau M, Antonenko S (1997). A CD4+ T-cell subset inhibits antigen-specific T-cell responses and prevents colitis.. Nature.

[51] Awasthi A, Carrier Y, Peron JP, Bettelli E, Kamanaka M (2007). A dominant function for interleukin 27 in generating interleukin 10-producing anti-inflammatory T cells.. Nat Immunol.

[52] Stumhofer JS, Silver JS, Laurence A, Porrett PM, Harris TH (2007). Interleukins 27 and 6 induce STAT3-mediated T cell production of interleukin 10.. Nat Immunol.

[53] Fitzgerald DC, Zhang GX, El-Behi M, Fonseca-Kelly Z, Li H (2007). Suppression of autoimmune inflammation of the central nervous system by interleukin 10 secreted by interleukin 27-stimulated T cells.. Nat Immunol.

[54] Akira S, Uematsu S, Takeuchi O (2006). Pathogen recognition and innate immunity.. Cell.

[55] Karimi K, Inman MD, Bienenstock J, Forsythe P (2009). Lactobacillus reuteri-induced regulatory T cells protect against an allergic airway response in mice.. Am J Respir Crit Care Med.

[56] Smits HH, Engering A, van der Kleij D, de Jong EC, Schipper K (2005). Selective probiotic bacteria induce IL-10-producing regulatory T cells in vitro by modulating dendritic cell function through dendritic cell-specific intercellular adhesion molecule 3-grabbing nonintegrin.. J Allergy Clin Immunol.

[57] Shima T, Fukushima K, Setoyama H, Imaoka A, Matsumoto S (2008). Differential effects of two probiotic strains with different bacteriological properties on intestinal gene expression, with special reference to indigenous bacteria.. FEMS Immunol Med Microbiol.

[58] Araki A, Kanai T, Ishikura T, Makita S, Uraushihara K (2005). MyD88-deficient mice develop severe intestinal inflammation in dextran sodium sulfate colitis.. J Gastroenterol.

[59] Rakoff-Nahoum S, Paglino J, Eslami-Varzaneh F, Edberg S, Medzhitov R (2004). Recognition of commensal microflora by toll-like receptors is required for intestinal homeostasis.. Cell.

[60] Rescigno M, Urbano M, Valzasina B, Francolini M, Rotta G (2001). Dendritic cells express tight junction proteins and penetrate gut epithelial monolayers to sample bacteria.. Nat Immunol.

[61] Fukuda S, Toh H, Hase K, Oshima K, Nakanishi Y (2011). Bifidobacteria can protect from enteropathogenic infection through production of acetate.. Nature.

[62] Habu Y, Nagaoka M, Yokokura T, Azuma I (1987). Structural studies of cell wall polysaccharides from Bifidobacterium breve YIT 4010 and related Bifidobacterium species.. J Biochem.

[63] Shida K, Nanno M (2008). Probiotics and immunology: separating the wheat from the chaff.. Trends Immunol.

[64] Kaji R, Kiyoshima-Shibata J, Nagaoka M, Nanno M, Shida K (2010). Bacterial teichoic acids reverse predominant IL-12 production induced by certain lactobacillus strains into predominant IL-10 production via TLR2-dependent ERK activation in macrophages.. J Immunol.

[65] Takeuchi O, Hoshino K, Kawai T, Sanjo H, Takada H (1999). Differential roles of TLR2 and TLR4 in recognition of gram-negative and gram-positive bacterial cell wall components.. Immunity.

[66] Yoshida H, Hamano S, Senaldi G, Covey T, Faggioni R (2001). WSX-1 is required for the initiation of Th1 responses and resistance to L. major infection.. Immunity.

[67] Atarashi K, Nishimura J, Shima T, Umesaki Y, Yamamoto M (2008). ATP drives lamina propria T(H)17 cell differentiation.. Nature.

[68] Kobayashi M, Kweon MN, Kuwata H, Schreiber RD, Kiyono H (2003). Toll-like receptor-dependent production of IL-12p40 causes chronic enterocolitis in myeloid cell-specific Stat3-deficient mice.. J Clin Invest.

