# Supplementary figures and images for: Probiotic Bifidobacterium breve Induces IL-10-Producing Tr1 Cells in the Colon
Source: PLoS Pathog. 2012 May 31;8(5):e1002714. doi: 10.1371/journal.ppat.1002714 (PMC3364948; doi:10.1371/journal.ppat.1002714)

Figure S1

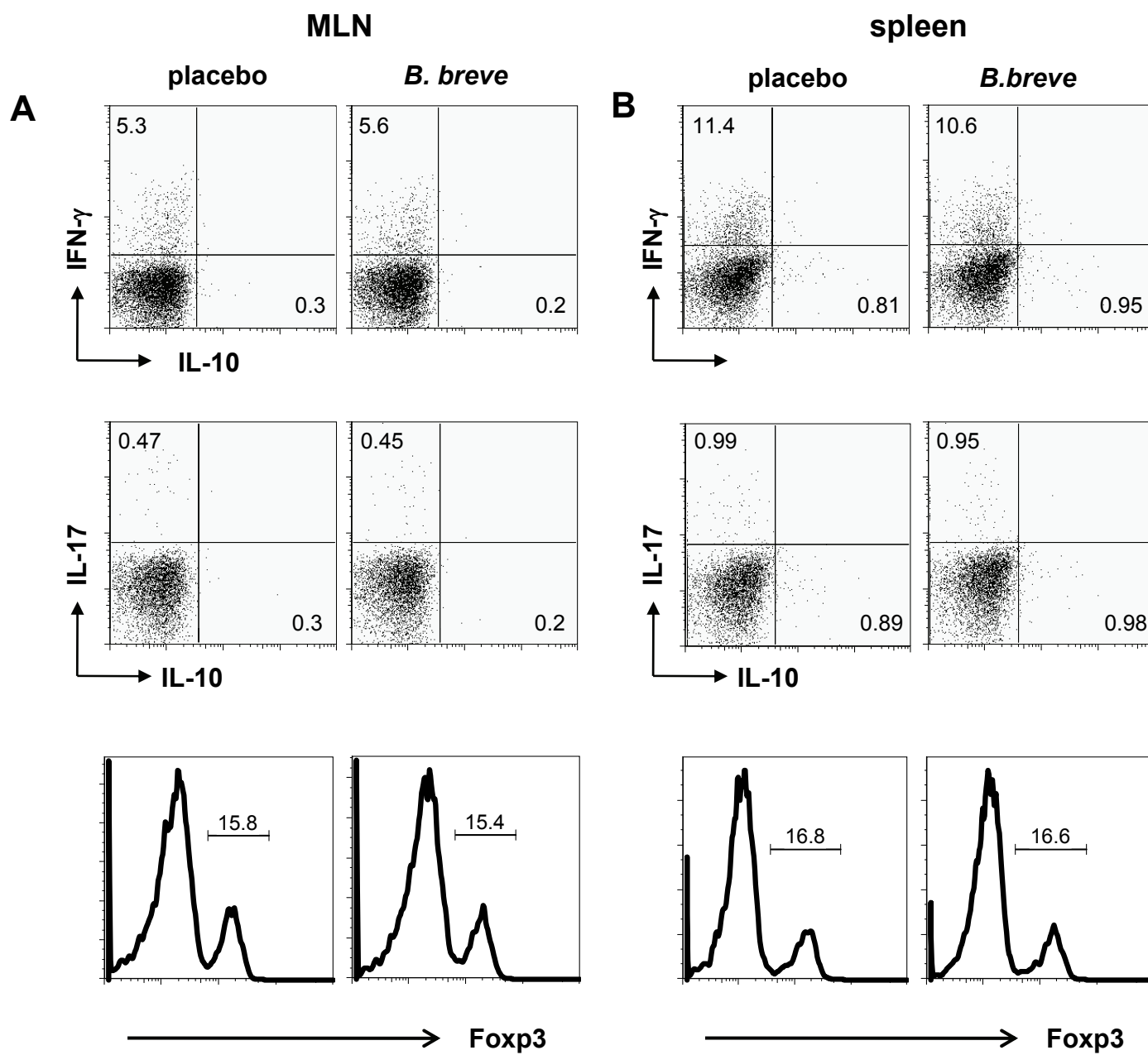

Supplement: Figure S1 — Percentage of IL-10+ or Foxp3+ CD4+ T cells in MLN or spleens were not changed by oral treatment of B. breve . 6-week-old C57BL/6 mice were fed with B. breve or placebo daily by oral gavage for 3 months (n = 8). MLNs and spleens were taken, and analyzed for expression of cytokines and Foxp3 by flow cytometry. Representative FACS dot plots were shown gated on CD4+ T cells. A: MLN, B: Spleen. (PDF) [file ppat.1002714.s001.pdf]

**Figure S2**

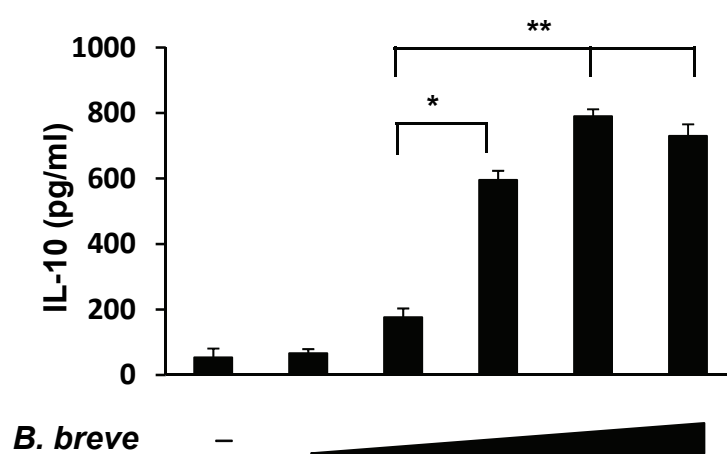

Supplement: Figure S2 — B. breve induces IL-10-producing Tr1 cells in a dose-dependent manner. CD11chigh CD11b−CD103+ DCs (CD103+ DCs) (5×10^4^) were isolated from the colonic lamina propria of C57BL/6J mice, and treated with the increasing numbers of B. breve (5×101 to 5×10^5^) for 24 h in round-bottom 96-well plate. After washing, splenic naïve CD4+ T cells (5×10^4^) were co-cultured with B.breve-treated CD103+ DC in the presence of anti-CD3 mAb for 4 days. Then, T cells were harvested and re-stimulated. IL-10 production in the culture supernatants was analyzed by ELISA. Data are representative of two independent experiments. Error bars, S.D. *P<0.05, **P<0.01. (PDF) [file ppat.1002714.s002.pdf]

**Figure S3**

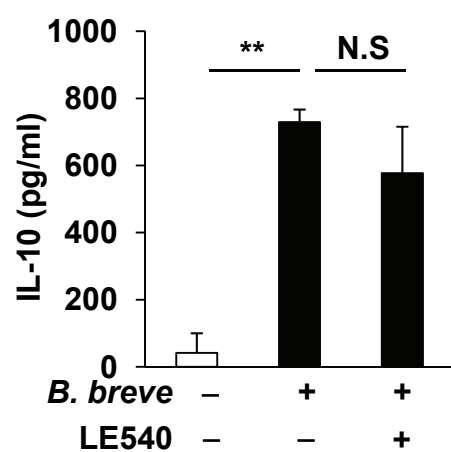

Supplement: Figure S3 — Retinoic acid-independent induction of Tr1 cells by B. breve . B. breve-treated CD103+ DCs were co-cultured with splenic naïve CD4+ T cells in the presence of an inhibitor of retinoic acid receptor (2 µM of LE540, WAKO chemicals, JAPAN) for 4 days. IL-10 production by re-stimulated T cells was quantified by ELISA. Data are representative of two independent experiments. Error bars, S.D. *P<0.01, N.S, not significant. (PDF) [file ppat.1002714.s003.pdf]

Figure S4

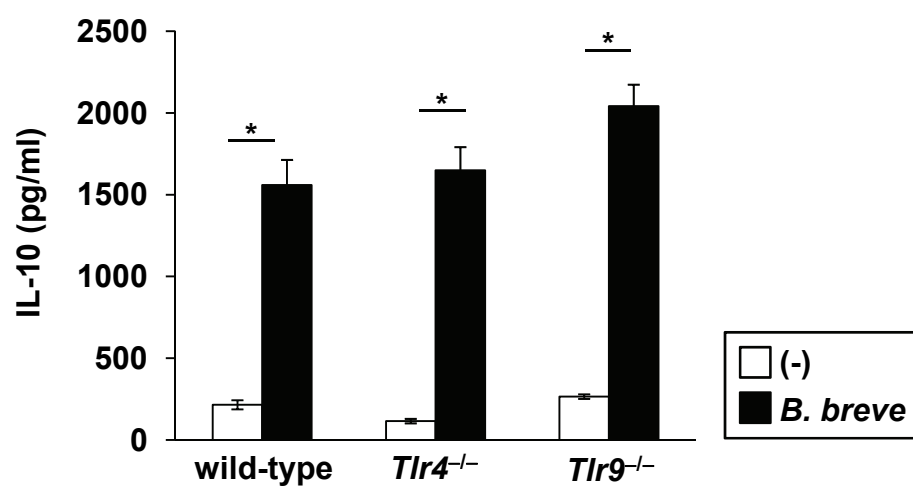

Supplement: Figure S4 — TLR4/TLR9-independent induction of Tr1 cells by B. breve . Intestinal CD103+ DCs from wild-type, Tlr4 −/− and Tlr9 −/− mice were treated with B. breve for 24 h, and then co-cultured with splenic naïve CD4+ T cells for 4 days. IL-10 production by re-stimulated T cells was quantified by ELISA. Data are representative of two independent experiments. Error bars, S.D. *P<0.01. (PDF) [file ppat.1002714.s004.pdf]

**Figure S5**

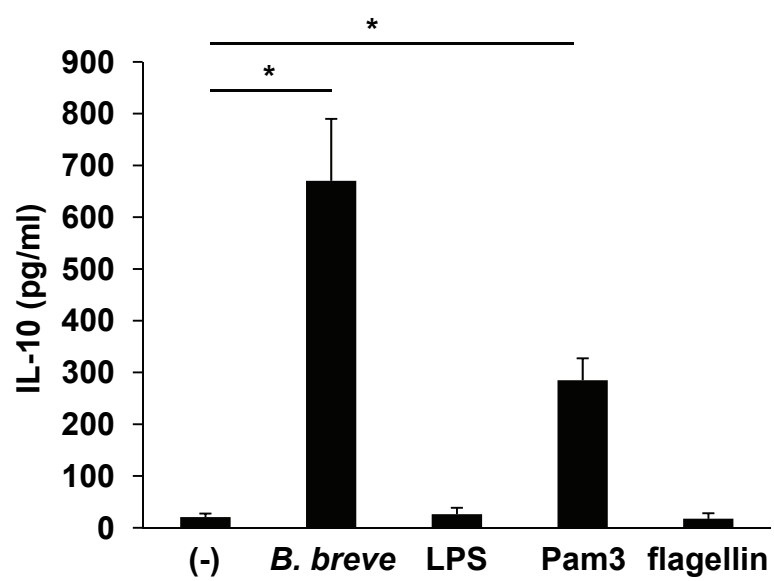

Supplement: Figure S5 — TLR2-dependent induction of Tr1 cells. Intestinal CD103+ DCs were stimulated with B. breve or TLR ligands such as LPS (TLR4 ligand), Pam3 (TLR2 ligand) or flagellin (TLR5 ligand) for 24 h, and then co-cultured with splenic naïve CD4+ T cells for 4 days. IL-10 production by re-stimulated T cells was quantified by ELISA. Data are representative of two independent experiments. Error bars, S.D. *P<0.01. (PDF) [file ppat.1002714.s005.pdf]

**Figure S6**

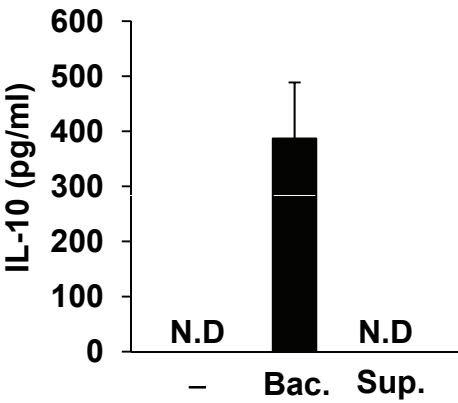

Supplement: Figure S6 — B. breve directly acts on CD103+ DCs to induce Tr1 cells. CD103+ DCs were treated by B.breve or culture supernatant (10-fold concentrated) of B. breve for 24 h. After washing, naïve CD4+ T cells were co-cultured with treated CD103+ DCs for 4 days. Then, T cells were harvested and re-stimulated by anti-CD3 and anti-CD28 mAbs. IL-10 concentration in the supernatants was quantified by ELISA. Representative data were shown from two independent experiments. Error bars, S.D. N.D, not detected. (PDF) [file ppat.1002714.s006.pdf]

**Figure S7**

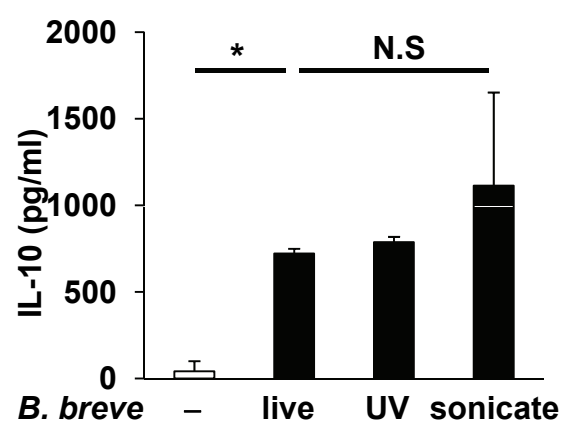

Supplement: Figure S7 — Induction of Tr1 cell development by killed B. breve . CD103+ DCs were treated by live, UV killed or sonicated B. breve for 24 h, and then, co-cultured with naïve CD4+ T cells for 4 days. T cells were harvested and re-stimulated by anti-CD3 and anti-CD28 mAbs. IL-10 concentration in the supernatants was quantified by ELISA. Data were representative of three independent experiments. Error bars, S.D. *P<0.01, N.S, not significant. (PDF) [file ppat.1002714.s007.pdf]
