# Supplementary material for: Probiotic Bifidobacterium breve Induces IL-10-Producing Tr1 Cells in the Colon
Source: PLoS Pathog. 2012 May 31;8(5):e1002714. doi: 10.1371/journal.ppat.1002714 (PMC3364948; doi:10.1371/journal.ppat.1002714)
Supplement: Table S1 — Composition of fecal commensal microflora in probiotics-fed mice. 6-week-old C57BL/6 mice were fed with L. casei, B. breve or placebo daily (1×10^9^) by oral gavage for 3 months (n = 5, respectively). Fecal samples were collected, weighed and suspended in 9 volumes of sterilized anaerobic transfer medium. Total RNA and DNA fractions extracted from each sample were assessed by RT-qPCR or qPCR with the specific primers. “Number” indicates CFU of each bacteria calculated using control cultured bacteria. (x/5) indicated the right side of “number” show detection rate of mice analyzed. (PDF) [file ppat.1002714.s008.pdf]

Table S1

bacterial species	non-treated		*L. casei*		placebo (*L. casei*)		*B. breve*		placebo (*B. breve*)	
	number		number		number		number		number	
**Total bacteria**	10.1 ±0.2	(5/5)	10.2 ±0.3	(5/5)	10.1 ±0.3	(5/5)	10.1 ±0.2	(5/5)	10.2 ±0.3	(5/5)
**Obligate anaerobic bacteria**										
Clostridium coccoides group	9.7 ±0.3	(5/5)	9.8 ±0.5	(5/5)	9.7 ±0.6	(5/5)	9.8 ±0.4	(5/5)	9.6 ±0.4	(5/5)
C. leptum subgroup	8.6 ±0.5	(5/5)	8.7 ±0.1	(5/5)	8.7 ±0.2	(5/5)	8.5 ±0.5	(5/5)	8.7 ±0.3	(5/5)
Bacteroides fragilis group	8.7 ±0.4	(5/5)	8.8 ±0.2	(5/5)	8.7 ±0.1	(5/5)	8.6 ±0.2	(5/5)	8.8 ±0.2	(5/5)
Total *Bifidobacterium*	7.5 ±0.5	(5/5)	7.5 ±0.7	(5/5)	7.6 ±0.4	(5/5)	7.6 ±0.3	(5/5)	7.6 ±0.4	(5/5)
Atopobium cluster	7.6 ±0.5	(5/5)	7.6 ±0.6	(5/5)	7.9 ±0.5	(5/5)	7.7 ±0.5	(5/5)	7.6 ±0.5	(5/5)
Prevotella	8.5 ±0.3	(5/5)	8.5 ±0.6	(5/5)	8.6 ±0.4	(5/5)	8.5 ±0.7	(5/5)	8.6 ±0.5	(5/5)
Eubacterium cylindroides group	7.7 ±0.6	(5/5)	7.5 ±0.3	(5/5)	7.7 ±0.3	(5/5)	7.6 ±0.3	(5/5)	7.6 ±0.4	(5/5)
C. ramosum subgroup	6.8 ±0.3	(5/5)	6.8 ±0.1	(5/5)	6.8 ±0.5	(5/5)	6.9 ±0.4	(5/5)	6.7 ±0.3	(5/5)
C. difficile	<2.1	(0/5)	<2.1	(0/5)	<2.1	(0/5)	5.0	(1/5)	<2.1	(0/5)
C. perfringens	<3.0	(0/5)	<3.0	(0/5)	<3.0	(0/5)	<3.0	(0/5)	<3.0	(0/5)
**Facultative anaerobic bacteria**										
Total *Lactobacillus*	9.7 ±0.2	(5/5)	9.7 ±0.1	(5/5)	9.6 ±0.1	(5/5)	9.3 ±0.1	(5/5)	9.8 ±0.5	(5/5)
L. gasseri subgroup	8.7 ±0.2	(5/5)	8.7 ±1.2	(5/5)	8.7 ±0.2	(5/5)	8.6 ±0.1	(5/5)	8.9 ±0.2	(5/5)
L. brevis	3.8 ±0.1	(4/5)	3.9 ±0.3	(4/5)	3.8 ±0.1	(5/5)	3.8 ±0.1	(4/5)	4.0 ±0.3	(5/5)
L. casei subgroup	<3.0	(0/5)	6.0 ±0.5	(5/5)	<3.0	(0/5)	<3.0	(0/5)	<3.0	(0/5)
L. fermentum	<3.9	(0/5)	<3.9	(0/5)	<3.9	(0/5)	<3.9	(0/5)	<3.9	(0/5)
L. fructivorans	<2.0	(0/5)	<2.0	(0/5)	<2.0	(0/5)	<2.0	(0/5)	<2.0	(0/5)
L. plantarum subgroup	3.1 ±0.1	(4/5)	3.2 ±0.8	(4/5)	3.0 ±0.1	(4/5)	3.1 ±0.6	(4/5)	3.0 ±0.2	(5/5)
L. reuteri subgroup	8.5 ±0.3	(5/5)	6.6 ±0.7	(5/5)	6.6 ±0.5	(5/5)	8.6 ±0.3	(5/5)	8.5 ±0.4	(5/5)
L. ruminis subgroup	9.6 ±0.2	(5/5)	9.5 ±0.1	(5/5)	9.5 ±0.2	(5/5)	9.5 ±0.1	(5/5)	9.6 ±0.6	(5/5)
L. sakei subgroup	5.4 ±0.3	(5/5)	5.1 ±0.2	(5/5)	5.3 ±0.3	(5/5)	5.5 ±0.4	(5/5)	5.3 ±0.6	(5/5)
Enterobacteriaceae	6.5 ±0.4	(5/5)	6.3 ±0.6	(5/5)	6.4 ±0.3	(5/5)	6.2 ±0.2	(5/5)	6.3 ±0.8	(5/5)
Enterococcus	8.6 ±0.2	(5/5)	8.4 ±0.1	(5/5)	8.4 ±0.2	(5/5)	8.4 ±0.2	(5/5)	8.5 ±0.5	(5/5)
Staphylococcus	5.1 ±0.6	(5/5)	5.1 ±0.5	(5/5)	4.9 ±0.4	(5/5)	5.0 ±0.1	(5/5)	4.9 ±0.5	(5/5)
**Aerobic bacteria**										
Pseudomonas	<3.7	(0/5)	<3.7	(0/5)	<3.7	(0/5)	<3.7	(0/5)	<3.7	(0/5)
**Administered probitic bacteria**										
L. casei strain Shirota	<6.0	(0/5)	6.3 ± 0.4	(3/5)	<6.0	(0/5)	<3.7	(0/5)	<6.0	(0/5)
B. breve Yakult strain	<6.0	(0/5)	<6.0	(0/5)	<6.0	(0/5)	6.3 ±0.3	(5/5)	<6.0	(0/5)

Table S1. Composition of fecal commensal microflora in probiotics-fed mice. 6-week-old C57BL/6 mice were fed with *L. casei*, *B. breve* or placebo daily (1 x 10^9) by oral gavage for 3 months (n=5, respectively). Fecal samples were collected, weighed and suspended in 9 volumes of sterilized anaerobic transfer medium. Total RNA and DNA fractions extracted from each sample were assessed by RT-qPCR or qPCR with the specific primers. "Number" indicates CFU of each bacteria calculated using control cultured bacteria. (x/5) indicated the right side of "number" show detection rate of mice analyzed.
