# Supplementary material for: Probiotic Bifidobacterium breve Induces IL-10-Producing Tr1 Cells in the Colon
Source: PLoS Pathog. 2012 May 31;8(5):e1002714. doi: 10.1371/journal.ppat.1002714 (PMC3364948; doi:10.1371/journal.ppat.1002714)
Supplement: Text S1 — Supplemental methods. Methods for “Analysis of Fecal Microbiota” and “Culture and Killing of B. breve” are described with references. (PDF) [file ppat.1002714.s009.pdf]

Supplementary Methods

Analysis of Fecal Microbiota

C57BL/6J mice were fed with *L. casei, B. breve* (10^9 bacteria each) or placebo daily by oral gavage for 3 months. Then, a few fecal pellets were collected, weighed and suspended in 9 volumes of RNA*later* (Ambion), an RNA stabilization solution, to make a fecal homogenate (100 mg feces/ml). In preparation for RNA and DNA extraction, 200 µl of the fecal homogenate was centrifuged and washed with PBS. The pellet was stored at -80°C (for extraction of RNA) or -30°C (for extraction of DNA). Total RNA and DNA for analysis fecal microflora (Matsuda et al., 2009), DNA for analysis of *L. casei* and *B. breve* (Fujimoto et al., 2008; Fujimoto et al., 2011) were isolated from fecal samples using the method described elsewhere. Briefly, total RNA was isolated using a modified acidic guanidinium thiocyanate-phenol-chloroform extraction method, and DNA was isolated using a modified phenol- chloroform extraction method. The DNA for analysis of *L. casei* and *B. breve* was extracted using a Stool Mini Kit (QIAGEN). Finally, the nucleic acid fraction extracted from 20 mg feces was suspended in 1 ml nuclease-free water (Ambion).

RT-qPCR and qPCR analyses were performed in 384-well optical plates on an ABI PRISM® 7900HT Sequence Detection System (Applied Biosystems). RT-qPCR was conducted in a one-step reaction using a QIAGEN OneStep RT-PCR Kit (QIAGEN) for detection and enumeration of the following bacterial targets: *C. difficile*, *C. perfringens*, Total *Lactobacillus* spp. (*L. gasseri* subgroup, *L. brevis*, *L. casei* subgroup, *L. fermentum*, *L. fructivorans*, *L. plantarum* subgroup, *L. reuteri* subgroup, *L. ruminis* subgroup, *L. sakei* subgroup), Enterobacteriaceae, *Enterococcus* spp., *Streptococcus* spp., *Staphylococcus* spp., and *Pseudomonas* spp. as described elswhere (Matsuda et al., 2007; Matsuda et al., 2009; Sakaguchi et al.). qPCR was carried out using rTaq (TaKaRa Bio) for detection and enumeration of the following bacterial targets: *Clostridium coccoides* group, *C. leptum* subgroup, *Bacteroides fragilis* group, Total

Bifidobacterium spp. (*B. adolescentis*, *B. angulatum*, *B. bifidum*, *B. breve*, *B. catenulatum* group, *B. longum*, *B. infantis*, *B. dentium*), *Atopobium* cluster, *Prevotella* spp., *Eubacterium cylindroides* group, *C. ramosum* subgroup, *L. casei* strain Shirota and *B. breve* strain Yakult as described previously (Fujimoto et al., 2008; Fujimoto et al., 2011; Matsuki et al., 2002; Matsuki et al., 2004; Matsuki et al., 1999; Matsuki et al., 1998).

Culture and killing of B. breve

B. breve was inoculated in GAM broth (Nissui Pharmaceutical) supplemented with 1%(w/v) glucose, and cultured for 24 h at 37 °C under anaerobic conditions. The supernatant of the culture medium was obtained by centrifugation at 15,000 rpm for 10 min, and concentrated to 10-fold by Centricon YM-3 (Milipore, USA). For killing of *B. breve*, UV radiation and sonication was used. For UV radiation, 5 ml of *B.breve* suspension was placed to petri dish, and then UV radiation was performed for 30 min by using 200 nm UV lamp. For sonication, total 5 min of sonication was performed with interval at every 1 min. Viability of *B.breve* was checked by MRS agar plate.

Supplementary References

Fujimoto, J., Matsuki, T., Sasamoto, M., Tomii, Y., and Watanabe, K. (2008). Identification and quantification of Lactobacillus casei strain Shirota in human feces with strain-specific primers derived from randomly amplified polymorphic DNA. Int J Food Microbiol *126*, 210-215.

Fujimoto, J., Tanigawa, K., Kudo, Y., Makino, H., and Watanabe, K. (2011). Identification and quantification of viable Bifidobacterium breve strain Yakult in human faeces by using strain-specific primers and propidium monoazide. J Appl Microbiol *110*, 209-217.

Matsuda, K., Tsuji, H., Asahara, T., Kado, Y., and Nomoto, K. (2007). Sensitive quantitative detection of commensal bacteria by rRNA-targeted reverse transcription-PCR. Appl Environ Microbiol *73*, 32-39.

Matsuda, K., Tsuji, H., Asahara, T., Matsumoto, K., Takada, T., and Nomoto, K. (2009).

Establishment of an analytical system for the human fecal microbiota, based on reverse transcription-quantitative PCR targeting of multicopy rRNA molecules. Appl Environ Microbiol *75*, 1961-1969.

Matsuki, T., Watanabe, K., Fujimoto, J., Miyamoto, Y., Takada, T., Matsumoto, K., Oyaizu, H., and Tanaka, R. (2002). Development of 16S rRNA-gene-targeted group-specific primers for the detection and identification of predominant bacteria in human feces. Appl Environ Microbiol *68*, 5445-5451.

Matsuki, T., Watanabe, K., Fujimoto, J., Takada, T., and Tanaka, R. (2004). Use of 16S rRNA gene-targeted group-specific primers for real-time PCR analysis of predominant bacteria in human feces. Appl Environ Microbiol *70*, 7220-7228.

Matsuki, T., Watanabe, K., Tanaka, R., Fukuda, M., and Oyaizu, H. (1999). Distribution of bifidobacterial species in human intestinal microflora examined with 16S rRNA-gene-targeted species-specific primers. Appl Environ Microbiol *65*, 4506-4512.

Matsuki, T., Watanabe, K., Tanaka, R., and Oyaizu, H. (1998). Rapid identification of human intestinal bifidobacteria by 16S rRNA-targeted species- and group-specific primers. FEMS Microbiol Lett *167*, 113-121.

Sakaguchi, S., Saito, M., Tsuji, H., Asahara, T., Takata, O., Fujimura, J., Nagata, S., Nomoto, K., and Shimizu, T. Bacterial rRNA-targeted reverse transcription-PCR used to identify pathogens responsible for fever with neutropenia. J Clin Microbiol *48*, 1624-1628.
